# Computationally restoring the potency of a clinical antibody against Omicron

**DOI:** 10.1038/s41586-024-07385-1

**Published:** 2024-05-08

**Authors:** Thomas A. Desautels, Kathryn T. Arrildt, Adam T. Zemla, Edmond Y. Lau, Fangqiang Zhu, Dante Ricci, Stephanie Cronin, Seth J. Zost, Elad Binshtein, Suzanne M. Scheaffer, Bernadeta Dadonaite, Brenden K. Petersen, Taylor B. Engdahl, Elaine Chen, Laura S. Handal, Lynn Hall, John W. Goforth, Denis Vashchenko, Sam Nguyen, Dina R. Weilhammer, Jacky Kai-Yin Lo, Bonnee Rubinfeld, Edwin A. Saada, Tracy Weisenberger, Tek-Hyung Lee, Bradley Whitener, James B. Case, Alexander Ladd, Mary S. Silva, Rebecca M. Haluska, Emilia A. Grzesiak, Christopher G. Earnhart, Svetlana Hopkins, Thomas W. Bates, Larissa B. Thackray, Brent W. Segelke, Tri-lab COVID-19 Consortium, Emily Z. Alipio Lyon, Penelope S. Anderson, Aram Avila-Herrera, William F. Bennett, Feliza A. Bourguet, Julian C. Chen, Matthew A. Coleman, Nicole M. Collette, Anastasiia Davis, Byron D. Vannest, Erika J. Fong, Sean Gilmore, Andre R. Goncalves, Sara B. Hall, Brooke Harmon, Wei He, Steven A. Hoang-Phou, Mikel Landajuela, Ted A. Laurence, Tek Hyung Lee, Felipe Leno Da Silva, Chao Liu, Terrel N. Mundhenk, Mariam V. Mohagheghi, Peter R. McIlroy, Le Thanh Mai Pham, Joseph C. Sanchez, Anupama Sinha, Emilia A. Solomon, Nicholas Watkins, Jiachen Yang, Congwang Ye, Boya Zhang, Antonietta Maria Lillo, Shivshankar Sundaram, Jesse D. Bloom, Michael S. Diamond, James E. Crowe, Robert H. Carnahan, Daniel M. Faissol

**Affiliations:** 1 Computational Engineering Division, Lawrence Livermore National Laboratory, Livermore, CA USA; 2 Biosciences and Biotechnology Division, Lawrence Livermore National Laboratory, Livermore, CA USA; 3 Global Security Computing Applications Division, Lawrence Livermore National Laboratory, Livermore, CA USA; 4 Vanderbilt Vaccine Center, Vanderbilt University Medical Center, Nashville, TN USA; 5 Department of Medicine, Washington University School of Medicine, St. Louis, MO USA; 6 Basic Sciences Division and Computational Biology Program, Fred Hutchinson Cancer Center, Seattle, WA USA; 7 Applications Simulations and Quality Division, Lawrence Livermore National Laboratory, Livermore, CA USA; 8 Joint Program Executive Office for Chemical, Biological, Radiological, and Nuclear Defense, US Department of Defense, Frederick, MD USA; 9 Joint Rsearch and Development Inc., Stafford, VA USA; 10 Los Alamos National Laboratory, Bioscience Division, Los Alamos, NM USA; 11 Center for Bioengineering, Lawrence Livermore National Laboratory, Livermore, CA USA; 12 Howard Hughes Medical Institute, Seattle, WA USA; 13 Molecular Microbiology, Washington University School of Medicine, St. Louis, MO USA; 14 Pathology and Immunology, Washington University School of Medicine, St. Louis, MO USA; 15 Department of Pediatrics, Vanderbilt University Medical Center, Nashville, TN USA; 16 Global Security Principal Directorate, Lawrence Livermore National Laboratory, Livermore, CA USA; 17 Biotechnology and Bioengineering, Sandia National Laboratories, Livermore, CA USA; 18 Materials Science Division, Lawrence Livermore National Laboratory, Livermore, CA USA; 19 Bioresource and Environmental Security, Sandia National Laboratories, Livermore, CA USA; 20 Present Address: Google, Alphabet Inc., Mountain View, CA USA; 21 Present Address: Vir Biotechnology, San Francisco, CA USA

**Keywords:** Protein design, SARS-CoV-2, Antibody therapy

## Abstract

The COVID-19 pandemic underscored the promise of monoclonal antibody-based prophylactic and therapeutic drugs^1–3^ and revealed how quickly viral escape can curtail effective options^4,5^. When the SARS-CoV-2 Omicron variant emerged in 2021, many antibody drug products lost potency, including Evusheld and its constituent, cilgavimab^4–6^. Cilgavimab, like its progenitor COV2-2130, is a class 3 antibody that is compatible with other antibodies in combination^4^ and is challenging to replace with existing approaches. Rapidly modifying such high-value antibodies to restore efficacy against emerging variants is a compelling mitigation strategy. We sought to redesign and renew the efficacy of COV2-2130 against Omicron BA.1 and BA.1.1 strains while maintaining efficacy against the dominant Delta variant. Here we show that our computationally redesigned antibody, 2130-1-0114-112, achieves this objective, simultaneously increases neutralization potency against Delta and subsequent variants of concern, and provides protection in vivo against the strains tested: WA1/2020, BA.1.1 and BA.5. Deep mutational scanning of tens of thousands of pseudovirus variants reveals that 2130-1-0114-112 improves broad potency without increasing escape liabilities. Our results suggest that computational approaches can optimize an antibody to target multiple escape variants, while simultaneously enriching potency. Our computational approach does not require experimental iterations or pre-existing binding data, thus enabling rapid response strategies to address escape variants or lessen escape vulnerabilities.

## Abstract (web summary)

By demonstrating a computational approach to restore the clinical efficacy of a COVID-19 antibody, the potential to rapidly update clinical antibodies is explored.

## Main

The COVID-19 pandemic has underscored the promise of monoclonal antibody-based drugs as prophylactic and therapeutic treatments for infectious disease. Multiple monoclonal antibody drug products that have demonstrated efficacy in preventing COVID-19 (ref. ^1^) were developed and authorized for emergency use by the US FDA, reducing deaths, hospitalization rates^2^ and reducing viral load^3^.

Despite these efforts, the SARS-CoV-2 variant Omicron BA.1 escaped many emergency-use monoclonal antibody and antibody combination drug products^6,7^. First reported in November 2021, BA.1 outcompeted all other variants of concern (VOCs) worldwide within weeks^8^. BA.1 contains over 50 substitutions, including 15 in the spike protein receptor-binding domain (RBD), the primary target for therapeutic and prophylactic antibodies. These substitutions reduce or eliminate the neutralization capacity of many authorized prophylactic and therapeutic antibodies^4,5,7^.

In particular, the antibody combination Evusheld—so far, the only antibody drug approved for pre-exposure prophylaxis in immunocompromised patients for whom vaccination is not always protective^1^—was overwhelmed by Omicron variants. Evusheld combines tixagevimab plus cilgavimab, which are derived from the progenitor monoclonal antibodies COV2-2196 and COV2-2130, respectively. The two-antibody cocktail exhibits 10–100-fold reduction in neutralizing potency against Omicron BA.1 compared with wild-type SARS-CoV-2 (refs. ^4,9^), but COV2-2130 lost approximately 1,000-fold neutralization potency against Omicron BA.1.1 compared with strains circulating earlier in the pandemic^7,10,11^.

COV2-2130 is a class 3 RBD-targeting antibody that blocks interaction between the RBD and human angiotensin-converting enzyme (ACE2) without competing with antibodies targeting the class 1 site on the RBD. Thus, class 1 and class 3 antibodies can be combined or co-administered for simultaneous binding and synergistic neutralization^12^. Although antibodies that target the class 3 site of the RBD have clear therapeutic utility in antibody combinations, the emergence of Omicron BA.1 and BA.1.1 undermined many antibodies currently available^4^. Furthermore, potently neutralizing antibodies targeting class 3 sites on the RBD are less frequently identified^12^, suggesting that they are more difficult to replace.

Computational redesign of a clinically proven monoclonal antibody shows promise for recovering efficacy against escape variants, especially for antibodies known to complement other monoclonal antibodies as part of a combination antibody drug product^12^. Thus, we sought to optimize COV2-2130 to restore potent neutralization of escape variants by introducing a small number of mutations in the paratope, then computationally assessing improvement to binding affinity. Our computational approach—generative unconstrained intelligent drug engineering (GUIDE)—combines high-performance computing, simulation and machine learning to co-optimize binding affinity to multiple antigen targets, such as RBDs from several SARS-CoV-2 strains, along with other critical attributes such as thermostability. The computational platform operates in a ‘zero-shot’ setting; that is, designs are created without iteration through, or input from, wet laboratory experiments on proposed antibody candidates, relatives or other derivatives of the parental antibody. Although more challenging, this zero-shot approach enables rapid production of antibody candidates optimized for multiple target antigens in response to exigencies presented by escape variants. Over a 3-week period, our computational platform repaired the activity of COV2-2130 against Omicron variants. The best-resulting antibody design introduces just four amino acid substitutions into COV2-2130, which could enable an immunobridging strategy in which the established efficacy and safety profile of the parental antibody is leveraged to enable an accelerated regulatory approval and enter clinical use more rapidly and at lower cost. Furthermore, this strategy may provide a rapid pathway for mitigating the threat of future viruses and their continually evolving mutations.

## Computational design

**Fig. 1:**
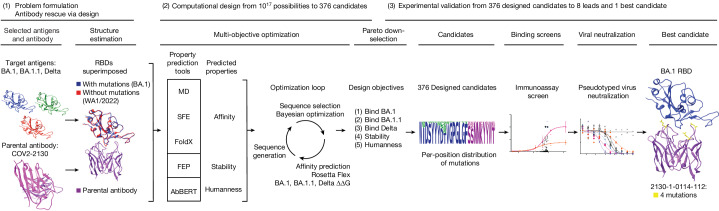
Application of the GUIDE computationally driven drug engineering platform to Omicron. Given a parental antibody and target antigens, a design space was defined and a collection of co-structures were estimated (left). Within the computational design phase (centre), a sequence generator used predictions of multiple properties to propose multi-point mutant antibody candidates, and a Bayesian optimization agent selected proposed sequences that were then simulated. On the basis of Pareto optimality, mutational distance and sequence diversity, 376 computationally evaluated sequences were selected and experimentally evaluated for binding in immunoassays (centre right). The best sequences were then evaluated for neutralization of SARS-CoV-2 variants, and the single best sequence was identified (right). See Supplementary Methods for details. FEP, free energy perturbation; MD, molecular dynamics; SFE, structural fluctuation estimation.

Our antibody design platform leverages simulation and machine learning to generate mutant antibody sequences that are co-optimized for multiple critical properties, without requiring experimental feedback or pre-existing binding data (Fig. 1). The platform comprises three phases: problem formulation, computational design and selection of mutant antibody candidates, and experimental validation of proposed candidates.

We formulated a problem by identifying a parental antibody, a set of target antigens and corresponding co-structures. In this case, we redesigned the COV2-2130 antibody^12^ for simultaneous binding improvements to Omicron BA.1 and BA.1.1 while maintaining binding to the Delta variant. We used co-structures that were both experimentally determined and computationally estimated, starting from co-structures including the wild-type antigen^13^. As an experimental structure of the Omicron RBD was not available at the onset of our design process, we estimated the structure of the complex of the RBD with COV2-2130 using template-based structural modelling^14^. We incorporated experimentally determined Omicron RBD structures^15^ as they became available. We considered 25 paratope residues for mutation, primarily in or near the heavy (H) or light (L) chain complementarity determining regions (CDRs)^16^, H2, H3, L1 and L2, and allowed up to 9 amino acid substitutions per mutant sequence, resulting in a search space containing over 10^17^ possible mutant sequences.

Our computational design approach was implemented as a multi-objective optimization problem defined over this large space of mutations to COV2-2130 paratope residues. We considered five critical antibody properties: (1) binding affinity to the Omicron BA.1 RBD, (2) binding affinity to the BA.1.1 RBD, (3) binding affinity to the Delta RBD, (4) thermostability, and (5) ‘humanness’. We expected that restored antibody affinity to each RBD variant would result in restored neutralization because the parental antibody, COV2-2130, competes with ACE2 in SARS-CoV-2 spike binding^12^. Four complementary computational tools enabled affinity prediction: atomistic potential of mean force molecular dynamics simulations, structural fluctuation estimation^17^, Rosetta Flex^18^ and FoldX^19^. We estimated thermal stability using the free energy perturbation method^20^. Humanness was quantified as the score under the AbBERT model^21^, a deep language model trained on a large database of human antibody sequences^22^. We used these tools to initialize a sequence generator, which proposed multi-residue mutations to the amino acid sequence of COV2-2130. Next, we used distributed software agents, each using Bayesian optimization or rules-based methods, to select a subset of promising candidate sequences to simulate in Rosetta Flex, yielding predicted binding affinities. In less than 3 weeks, we evaluated more than 125,000 antibody candidates.

We calculated the Pareto optimal set^23^ based on the outputs of these tools, resulting in 3,809 sequences. Owing to experimental capacity, we further downselected from among the Pareto set based on mutational distance and sequence diversity to ultimately designate 376 antibody sequences for experimental evaluation.

## Experimental evaluation

### Antibody and antigen production

We experimentally validated the 376 designed candidates. To leverage available resources at multiple experimental sites, we split candidates into partially overlapping sets 1 and 2. Set 1 consisted of 230 designs expressed as IgG in HEK-293 cells (ATUM), and set 2 consisted of 204 designs expressed as IgG via a pVVC-mCisK_hG1 vector (Twist BioScience) in transiently transfected CHO cells. Omicron antigens were produced in Expi293F cells (Thermo Fisher Scientific) and purified on HisTrap Excel columns (Cytiva).

In the following experiments, we selected antigens or viral strains to gauge the success of three goals: (1) improving binding affinity and efficacy to BA.1 and BA.1.1; (2) maintaining efficacy to historical strains, for which design explicitly targeted Delta but experiments often substituted WA1/2020 D614G; and (3) determining robustness to emerging VOCs.

### Designed antibodies maintain expression

Because in silico derivatization of antibody sequences can compromise production yield, we measured the concentrations of the 230 COV2-2130-derived recombinant antibodies in set 1 and compared these concentrations to that of the parental antibody. The purified concentrations of 73.9% of redesigned antibodies exceeded that of the parental COV2-2130 antibody (170 of 230 monoclonal antibodies at more than 171.2 mg l^−1^), reaching as high as 305 mg l^−1^. Our designed candidates for downstream characterization retained fundamental production properties of the parental antibody, with just 10% of designed antibodies producing poor yields relative to the parental molecule (22 of 230 monoclonal antibodies at less than 135 mg l^−1^, that is, less than 80% of the parental antibody yield).

### Thermostability and binding Omicron

**Extended Data Fig. 1:**
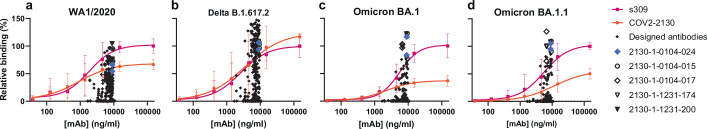
Optimized, single-concentration screening data in Gyrolab immunoassays allow selection of candidates from Set 1 (n = 230) for down-stream characterization. **a-d**, Parental mAb COV2-2130 (orange circles) and positive control mAb S309 24 (magenta squares) serve as references for computationally designed mAbs in single-concentration immunoassays. Computationally designed antibodies are shown as gray diamonds; selected computationally designed antibodies are highlighted with other colors and symbols as shown in the legend. Target antigens are (**a**) wild type WA1/2020, (**b**) Delta, (**c**) Omicron BA.1 and (**d**) Omicron BA.1.1. Each screened antibody and antigen combination was evaluated in two replicate assays, shown as individual points in the plot. Each of the controls was replicated in two replicate assays for each of three groups of antibodies, resulting in six replicate assays for each point on the control curves. Control curve points and error bars indicate mean and standard deviation. Curves represent a four-parameter logistic curve fit to the control data. All analysis performed in GraphPad Prism.

**Extended Data Fig. 2:**
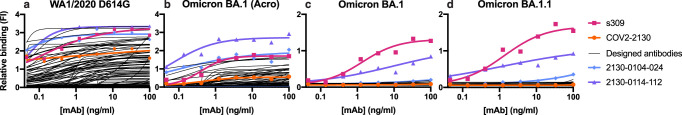
ELISA screening allows set for down selection of candidates from Set 2 (n = 204), for down-stream characterization. **a-d**, Parental mAb COV2-2130 (orange circles; 3 technical replicates) and positive control mAb S309 24 (magenta squares; 3 technical replicates) serve as references for computationally designed mAbs (black curves). Purple triangles are 2130-1-114-112; blue diamonds are 2130-1-0104-024. Each designed antibody had a single measurement (n = 1) at each concentration. All curves are 4-parameter logistic fits, produced in GraphPad Prism. Target antigens are RBD from (**a**) wild type WA1/2020, (**b**) Omicron BA.1 (Acro), which is biotinylated, (**c**) Omicron BA.1, and (**d**) Omicron BA.1.1. For the biotinylated antigen in (**b**) (Acro Biosystems, cat. SPD-C82E4), an additional coat and wash cycle was required to prepare the ELISA plate with streptavidin.

We screened all designed antibodies for binding to RBDs. Set 1 was screened via a single-concentration immunoassay (Gyrolab xPlore) in the contexts of WA1/2020, Delta, BA.1 or BA.1.1 RBDs (Extended Data Fig. 1). For set 2, we used a multi-concentration immunoassay (ELISA; Extended Data Fig. 2) in the context of wild-type, BA.1 or BA.1.1 RBDs. In the single-concentration immunoassay, this value was chosen as a single dilution factor, causing most designed antibody samples to fall in the dynamic range of the positive control. In both cases, we compared the binding of the designed antibodies with a broadly cross-reactive, non-ACE2-competitive control antibody (S309)^24^ and the parental COV2-2130 antibody. As intended, most antibody designs had altered binding profiles, indicating that the designed mutations were consequential. Approximately 11% of the designs of set 1 retained WA1/2020 antigen binding at the measured concentration; roughly 6% improved binding to BA.1, and 5% did so to BA.1.1. The corresponding numbers for set 2 were 9% to WA1/2020 and 8% to BA.1. Following this initial screen, we downselected both sets of antibody designs to those with improved binding to Omicron subvariants BA.1 and BA.1.1 for further characterization.

**Fig. 2:**
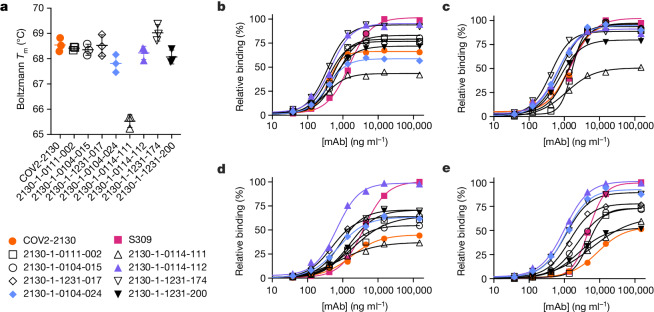
Computationally designed IgG antibodies improve Omicron binding and maintain parental thermostability and binding to historical strains. **a**, The parental COV2-2130 (orange circles) and computationally designed antibodies (2130-1-0114-112 in purple triangles, 2130-1-0104-024 in blue diamonds and remainder in black) were assayed for thermal shift (*n* = 3 technical replicates). Melting temperature (*T*_m_ ) calculated based on the Boltzmann method. Data are mean and s.d. **b**–**e**, The parental COV2-2130 antibody and computationally designed antibodies (see symbols in **a**) and cross-reactive positive control antibody S309 (magenta squares) were analysed for relative binding to four SARS-CoV-2 spike RBD variants in the Gyrolab immunoassay: WA1/2020 (**b**), Delta B.1.617.2 (**c**), Omicron BA.1 (**d**) and Omicron BA.1.1 (**e**). Lines represent a four-parameter logistic regression model fit using GraphPad Prism to each titration, executed without technical replicates. mAb, monoclonal antibody.

These downselected antibodies were re-manufactured at larger scale. We characterized the resulting IgG antibodies by immunoassay and thermal shift (melt temperature) assessments. In agreement with our screens, seven of the eight top-performing antibodies preserved comparable binding with WA1/2020 and Delta RBDs, improving over the parental COV2-2130 antibody with respect to their binding to Omicron BA.1 and BA.1.1 RBDs (Fig. 2). Furthermore, seven of the eight antibodies had melting temperatures and expression properties comparable with those of COV2-2130. One antibody, 2130-1-0114-111, had reduced melting temperature (Extended Data Table 1). The antibody 2130-1-0114-112 displayed best-in-class binding across all RBD variants and had no substantial difference in thermal stability compared with the parental COV2-2130 antibody.

### Restored pseudoviral neutralization

**Fig. 3:**
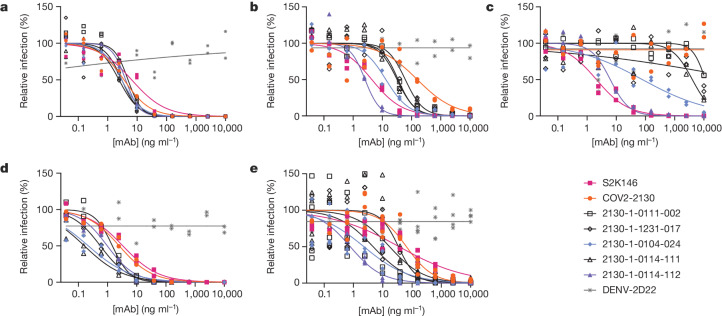
Designed antibodies improve pseudoviral neutralization over COV2-2130. **a**–**e**, The parental COV2-2130 antibody (orange circles), the cross-reactive positive control antibody S2K146 (magenta squares), the negative control antibody DENV-2D22 (grey x) and down-selected computationally designed antibodies (symbols as indicated in the key) were assayed by neutralization with lentiviruses pseudotyped with spike variants of WA1/2020 D614G (**a**), Omicron BA.1 (**b**), Omicron BA.1.1 (**c**), Omicron BA.2 (**d**) and Omicron BA.4 (**e**). Curves are four-parameter logistic regression models fit to two (**a**–**d**) or four (**e**) replicate serial dilutions using GraphPad Prism.

We performed pseudovirus neutralization assays to characterize the functional performance of five selected antibody designs (Fig. 3 and Extended Data Table 2), compared with parental COV2-2130; the positive control S2K146 (ref. ^25^), which competes with ACE2 binding; and the negative control DENV-2D22 (ref. ^26^). Our designs maintained neutralization activity against pseudoviruses displaying historical spike proteins (WA1/2020 D614G) and achieved neutralization of those with Omicron BA.1 spikes. The single-best candidate design, 2130-1-0114-112, restored potent neutralization in the context of BA.1.1 and showed a two-order-of-magnitude improvement in the half-maximal inhibitory concentration (IC_50_) versus parental COV2-2130 for BA.1 and BA.4. These pseudovirus neutralization test results showed that our designs neutralized BA.2 and BA.4 more potently than COV2-2130, despite the emergence of these VOCs after the conception of our designs.

### Restored authentic virus neutralization

**Extended Data Fig. 3:**
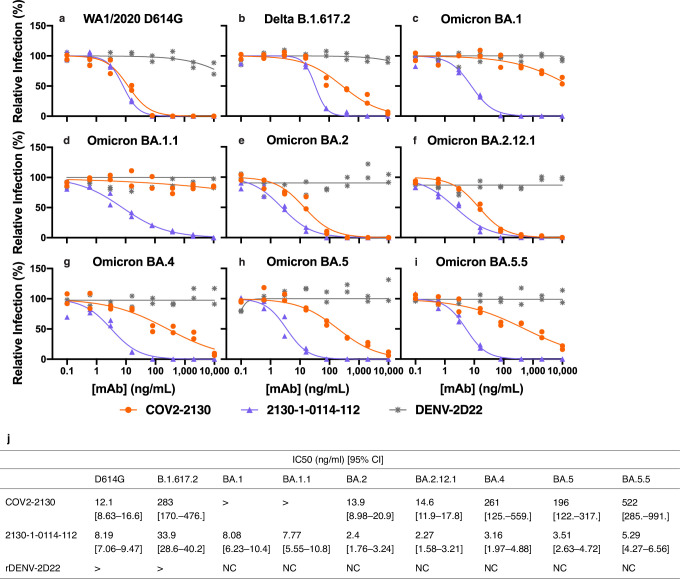
2130-1-0114-112 is potent in focus reduction neutralization tests with authentic virus in Vero-TMPRSS2 cells. **a-i**, 2130-1-0114-112 potently neutralizes (**a**) WA1/2020 D614G (**b**) Delta B.1.617.2, (**c**) Omicron BA.1, (**d**) Omicron BA.1.1, (**e**) Omicron BA.2, (**f**) Omicron BA.2.12.1, (**g**) Omicron BA.4, (**h**) Omicron BA.5, and (**i**) Omicron BA.5.5 authentic viruses in focus reduction neutralization assays in Vero-TMPRSS2 cells. Symbols indicate the values of two technical replicates; curves are 4-parameter logistic regression models fit of normalized data. **j**, IC50 values and 95% confidence intervals corresponding to **a-i**. “>” indicates IC50 values > 10,000; “NC” indicates fits that were unconverged, unstable, or with positive hill slope. Analyses were performed in GraphPad Prism.

**Extended Data Fig. 4:**
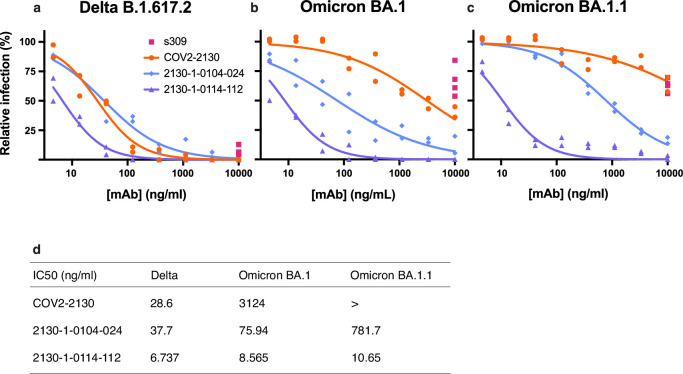
Authentic virus neutralization in plaque assays using Vero E6-TMPRSS2-T2A-ACE2 (VAT) cells. **a-c**, Plaque assay neutralization of (**a**) Delta, (**b**) Omicron BA.1 and (**c**) Omicron BA.1.1 viruses. Data are represented as the normalized infection of mAb-treated virus to virus treated with control human IgG (Invitrogen). For S309, each point shows one of four technical replicates; all other points show one of two technical replicates at each concentration. Curves are two-parameter (IC50, hill-slope) logistic fits to normalized response. **d**, IC50 values (ng/ml, “>” indicates IC50 greater than 10,000 ng/mL) show that 2130-1-0104-024, while having only two mutations from COV2-2130, remains potent against BA.1 and suffers a 20-times loss in potency against BA.1.1. 2130-1-0114-112 is strongly potent against all three tested variants. All analysis performed in GraphPad Prism.

We evaluated 2130-1-0114-112 (containing four mutations: GH112E, SL32A, SL33A and TL59E) for authentic virus neutralization performance against several strains of SARS-CoV-2 by a focus reduction neutralization test in Vero-TMPRSS2 cells (Extended Data Fig. 3). The strains that we used included several Omicron targets: WA1/2020 D614G, Delta (B.1.617.2), BA.1, BA.1.1, BA.2, BA.2.12.1, BA.4, BA.5 and BA.5.5. In all cases apart from Delta, 2130-1-0114-112 had an IC_50_ < 10 ng ml^−1^. Compared with the parental COV2-2130, 2130-1-0114-112 restored potent neutralization activity to both BA.1 (8.08 ng ml^−1^) and BA.1.1 (7.77 ng ml^−1^), showed a more than fivefold improvement in IC_50_ to BA.2 (2.4 ng ml^−1^) and BA.2.12.1 (2.27 ng ml^−1^), and conferred 50-fold or greater improvements in IC_50_ to BA.4 (3.16 ng ml^−1^), BA.5 (3.51 ng ml^−1^) and BA.5.5 (5.29 ng ml^−1^). We also evaluated 2130-1-0114-112 and a less-mutated alternative design, 2130-1-0104-024 (containing two mutations: SL32W and TL59E), in plaque assays with Vero E6-TMPRSS2-T2A-ACE2 cells (Extended Data Fig. 4). IC_50_ values for 2130-1-0104-024 were 37.7 ng ml^−1^, 75.94 ng ml^−1^ and 781.7 ng ml^−1^ for Delta, BA.1 and BA.1.1 viruses, respectively.

### Prophylaxis in vivo

**Fig. 4:**
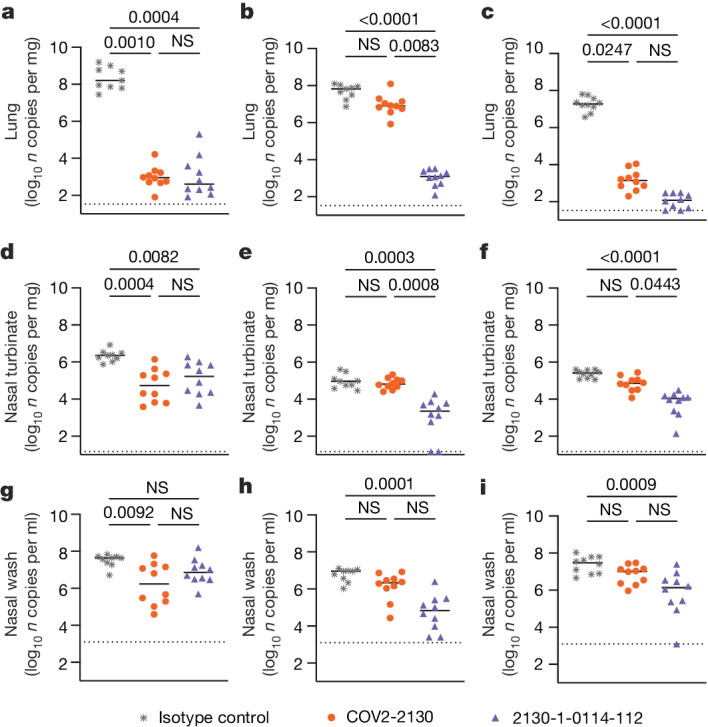
2130-1-0114-112 provides in vivo prophylactic protection against SARS-CoV-2 variants. **a**–**i**, Eight-week-old female K18-hACE2 mice were administered 100 μg (approximately 5 mg kg^−1^) of the indicated monoclonal antibody treatment by intraperitoneal injection 1 day before intranasal inoculation with 10^4^ focus-forming units (FFU) of WA1/2020 D614G (**a**,**d**,**g**), Omicron BA.1.1 (**b**,**e**,**h**) or Omicron BA.5 (**c**,**f**,**i**). Tissues were collected 4 days after inoculation. Viral RNA levels in the lungs (**a**–**c**), nasal turbinates (**d**–**f**) and nasal washes (**g**–**i**) were determined by RT–qPCR (lines indicate median of log_10_ values); *n*  =  9 (WA1/2020 D614G and BA.1.1 isotype control groups) or 10 (all others) mice per group, from two experiments. The limit of assay detection is shown as a horizontal dotted line. Statistical comparisons between groups were by Kruskal–Wallis ANOVA with Dunn’s multiple comparisons post-test; *P* values are as listed or not significant (NS) if *P* > 0.05. All analyses were conducted in GraphPad Prism. Source data

To compare the prophylactic efficacy of 2130-1-0114-112 and the parental COV2-2130 monoclonal antibody in vivo, we administered a single 100 μg (approximately 5 mg kg^−1^ total) dose to K18-hACE2 transgenic mice 1 day before intranasal inoculation with WA1/2020 D614G, BA.1.1 or BA.5 (88 mice in total, 9–10 for each monoclonal antibody and viral strain). Although Omicron viruses are less pathogenic in mice than in humans, they replicate efficiently in the lungs of K18-hACE2 mice^27,28^. Viral RNA levels were measured at 4 days post-infection in the nasal washes, nasal turbinates and lungs (Fig. 4). As expected, the parental COV2-2130 monoclonal antibody effectively reduced WA1/2020 D614G infection in the lungs (180,930-fold), nasal turbinates (42-fold) and nasal washes (25-fold) compared with the isotype control monoclonal antibody. However, the COV2-2130 monoclonal antibody lost protective activity to BA.1.1 in all respiratory tract tissues, whereas to BA.5, protection was maintained in the lungs (13,622-fold) but not in the nasal turbinates or nasal washes. Compared with the isotype control monoclonal antibody (Fig. 4), 2130-1-0114-112 protected against lung infection by WA1/2020 D614G (399,945-fold reduction), BA.1.1 (53,468-fold reduction) and BA.5 (160,133-fold reduction). Moreover, in the upper respiratory tract, 2130-1-0114-112 also conferred protection to WA1/2020 D614G, BA.1.1 and BA.5. The differences in protection between the parental COV2-2130 and derivative 2130-1-0114-112 monoclonal antibodies were most apparent in mice infected with BA.1.1, which directly parallels the neutralization data (Fig. 3 and Extended Data Figs. 3 and 4).

### Potency without additional liabilities

**Fig. 5:**
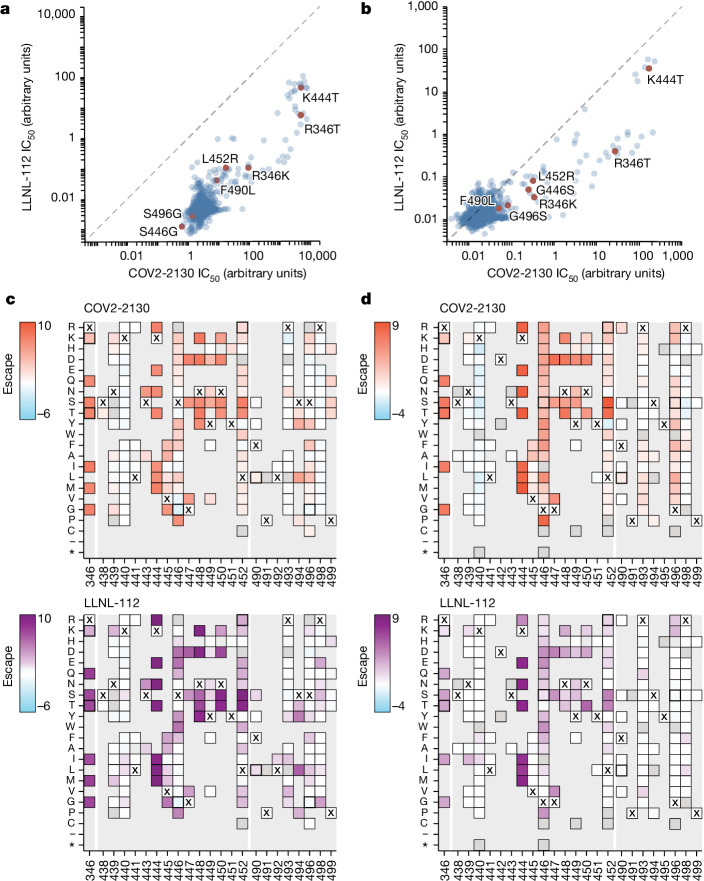
COV-2130 and 2130-1-0114-112 escape mapping using DMS. **a**,**b**, Comparison between IC_50_ values measured using DMS for COV-2130 and 2130-1-0114-112 antibodies in BA.1 (**a**) and BA.2 (**b**) backgrounds, with key mutations highlighted. Arbitrary units in both plots are on the same scale. Interactive plots that display each mutation can be found at https://dms-vep.org/SARS-CoV-2_Omicron_BA.1_spike_DMS_COV2-2130/compare_IC50s.html for the BA.1 background and at https://dms-vep.org/SARS-CoV-2_Omicron_BA.2_spike_DMS_COV2-2130/compare_IC50s.html for the BA.2 background. **c**,**d**, Heatmaps of mutation escape scores at key sites for each antibody in BA.1 (**c**) and BA.2 (**d**) backgrounds. Escape scores were calculated relative to the wild-type amino acid in the same virus background. X marks wild-type amino acid in the relevant background. Amino acids not present in the DMS libraries lack squares; grey squares are mutations that strongly impair spike-mediated infection. Mutations identified in **a**,**b** are shown with a heavy line surrounding the corresponding box in **c**,**d**. Interactive heatmaps for full spike can be found for the BA.1 background at https://dms-vep.org/SARS-CoV-2_Omicron_BA.1_spike_DMS_COV2-2130/COV2-2130_vs_2130-1-0114-112_escape.html and https://dms-vep.org/SARS-CoV-2_Omicron_BA.2_spike_DMS_COV2-2130/COV2-2130_vs_2130-1-0114-112_escape.html for the BA.2 background.

**Extended Data Fig. 5:**
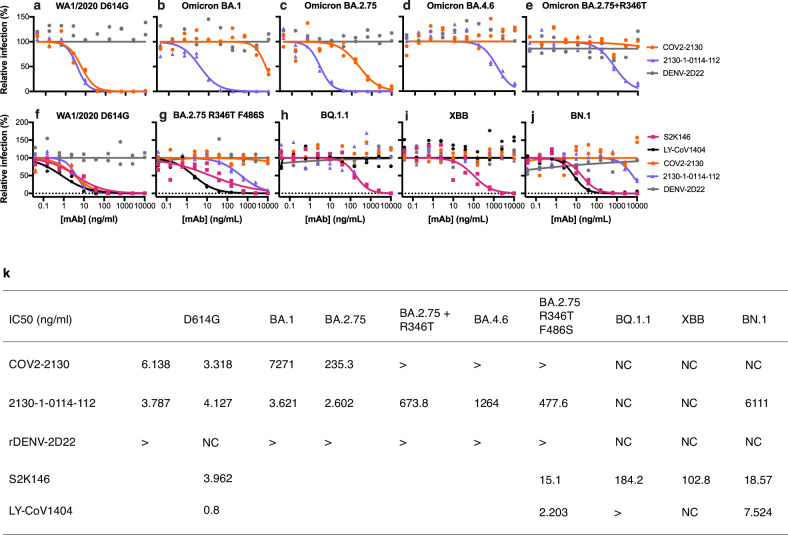
Normalized pseudoviral neutralization of SARS-CoV-2 VOCs by 2130-1-0114-112. **a, f**, COV2-2130 and 2130-1-0114-112 potently neutralize WA1/2020 D614G. **b**, COV2-2130 does not potently neutralize BA.1, whereas 2130-1-0114-112 does, consistent with other pseudoviral neutralization assays. **c**, 2130-1-0114-112 potently neutralizes BA.2.75, outperforming COV2-2130 by 90-fold. **d, e**, 2130-1-0114-112 loses substantial potency in the context of BA.4.6 and artificially-produced BA.2.75 + R346T but retains measurable neutralization, demonstrating mitigation of this critical weakness of COV2-2130. **g-j**, 2130-1-0114-112 maintains weak neutralization of BA.2.75 R346T F486S (**g**), but loses detectable neutralization activity against BQ.1.1 (**h**) and XBB (**i**) and exhibits a near-complete loss of neutralization activity against BN.1 (**j**). Symbols indicate two independent technical replicates at each concentration. Curves represent a four-parameter logistic curve fit to the data with fixed minimum and maximum values (0 and 100, respectively). **k**, Neutralization IC50 values in ng/ml. “>” indicates IC50 greater than 10,000 ng/mL. “NC” indicates failure to converge. All analysis conducted in GraphPad Prism.

To understand the neutralization breadth of 2130-1-0114-112 relative to its ancestral antibody, we mapped the epitopes for both antibodies using spike-pseudotyped lentiviral deep mutational scanning (DMS)^29^. For each antibody, we mapped escape mutations in both the BA.1 and the BA.2 spikes. DMS experiments showed that the escape profile of both COV2-2130 and 2130-1-0114-112 in the context of both BA.1 and BA.2 backgrounds is consistent with the epitope of the antibodies, but with differences in sensitivity to particular mutations (Fig. 5). Consistent with live and pseudovirus neutralization assays (Fig. 3 and Extended Data Figs. 3 and 4), mutations at RBD positions R346 and L452 are sites of substantial escape from both antibodies (Fig. 5). In addition, both antibodies lose potency with mutations at site K444 (such as K444T found in BQ.1* variants). The reversion mutation S446G in the BA.1 background increases the neutralization potency of both antibodies (negative escape values in heatmaps) (Fig. 5c) and probably contributes to greater neutralization potency to the BA.2 variant (Fig. 3 and Extended Data Fig. 3), which carries G446. Most mutations at RBD sites K440 and R498 are slightly sensitizing to the COV2-2130 antibody in both BA.1 and BA.2 backgrounds, but provide weak escape for 2130-1-0114-112 in the BA.1 background and have even weaker effect in the BA.2 background. In agreement with pseudovirus neutralization assays (Fig. 3), comparison of mutation-level escape showed that the 2130-1-0114-112 antibody is substantially more potent than COV2-2130 to the BA.1 variant and retains better potency against viruses with additional mutations in both BA.1 and BA.2 backgrounds (Fig. 5a,b). However, even with improved potency, 2130-1-0114-112 is still vulnerable to escape at multiple RBD residues in the 444–452 loop, which is the site of convergent substitutions in several Omicron lineages^30^. Many of these variants contain multiple substitutions at positions identified by DMS as important for neutralization or in close proximity to the COV2-2130 epitope, including BQ.1.1 (R346T and K444T), XBB (R346T, V445P and G446S) and BN.1 (R346T, K356T and G446S). To understand the impact of these VOCs, we assessed the ability of 2130-1-0114-112 to neutralize BQ.1.1, XBB and BN.1 in pseudoviral neutralization studies. Consistent with the previously known liabilities of COV2-2130 and our DMS results, 2130-1-0114-112 loses neutralizing activity to these VOCs (Extended Data Fig. 5), probably due to substitutions at 444 and combinatorial effects of multiple substitutions within the COV2-2130 epitope present in these variants. Together, these data demonstrate that 2130-1-0114-112 exhibits improved potency against many individual substitutions without incurring additional escape liabilities, although RBD residues such as 444 remain critical for neutralization activity of both 2130-1-0144-112 and COV2-2130.

#### Structural basis for restored potency

**Fig. 6:**
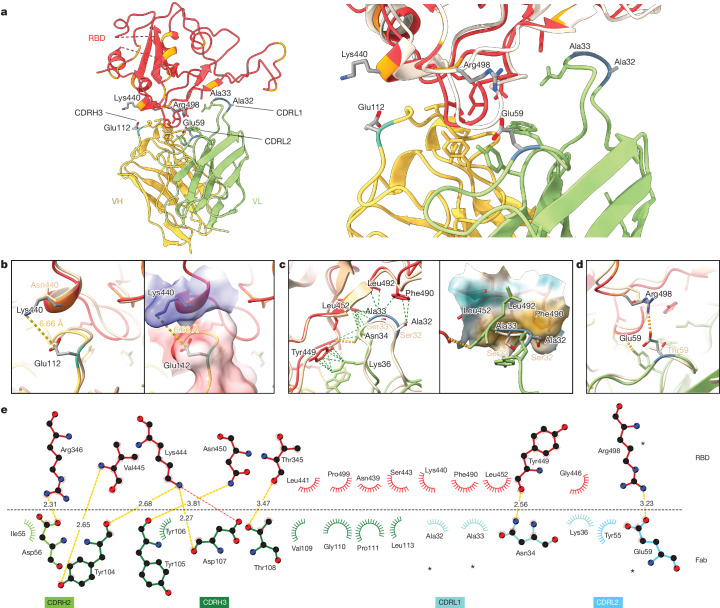
Cryo-EM structure of neutralizing antibodies 2130-1-0114-112 in complex with Omicron BA.2 RBD. **a**, Atomic model of the RBD–Fab complex, coloured by chain: BA.2 RBD in red, 2130-1-0114-112 HC in yellow and 2130-1-0114-112 LC in green. BA.2 RBD mutations are in orange, and 2130-1-0114-112 mutations are in cyan and blue (HC and LC) (left). A close-up view of the RBD–Fab interface, showing WA1 RBD (Protein Data Bank 7L7E, light brown shading) aligned with the BA.2 RBD (right). **b**–**d**, Details showing the 2130-1-0114-112 modified residues and their interaction with BA.2 RBD, coloured as in **a**. Residue labels are shown in black for the BA.2 complex and brown for the overlaid WA1-2130 complex. The orange and green dashed lines indicate hydrogen bond and hydrophobic interactions, respectively; the yellow dashed lines are labelled with distances. CDRH3 residue Glu112 (left) and with the surface coloured by electrostatic potential (right), showing the positive and negative charges of RBD Lys444 and CDRH3 Glu112 (**b**). CDRL1 Ala32 and Ala33 hydrophobic network (left) and with the nearby RBD surface coloured by hydrophobicity (right; orange to cyan indicates hydrophobic to hydrophilic) (**c**). CDRL2 Glu59 salt bridge with RBD residue Arg498 (**d**). **e**, 2D diagram of Fab 2130-1-0114-112 paratope and epitope residues involved in hydrogen bonds and salt bridges (yellow and red dashed lines, respectively; distances in Å) and hydrophobic interactions (curved lines with rays). Atoms are shown as circles, with oxygen, carbon and nitrogen in red, black and blue, respectively. Interacting residues that belong to CDR loops are coloured in corresponding shades. The asterisks indicate mutated residues. Image created with Ligplot+^34^.

**Extended Data Fig. 6:**
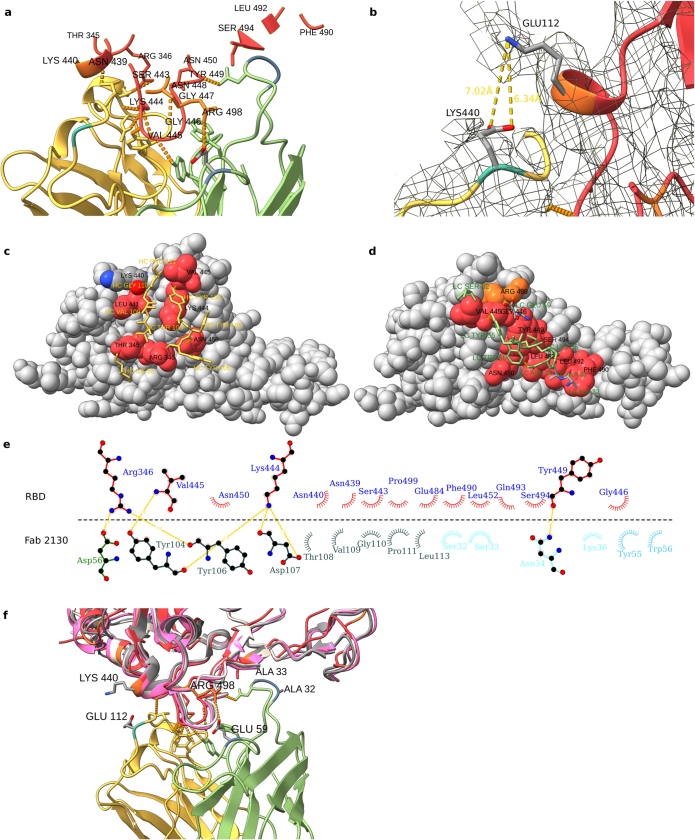
Details and VOC comparisons for Cryo-EM structure of neutralizing antibody 2130-1-0114-112 in complex with Omicron BA.2 RBD. **a**, RBD residues within 7 Å of 2130-1-0114-112. RBD shown in red, with BA.2 mutated residues in orange, and 2130-1-0114-112 in yellow/green. **b**, CDRH3 Glu112 and RBD Lys440, shown with the EM map and distance between the side chains. **c, d**, 3D representation of the interaction plot between RBD and 2130-1-0114-112 HC (**c**, yellow) and LC (**d**, green). 2130-1-0114-112 is shown as stick and RBD as gray spheres with the contact residues in red. Contact residues are labelled and numbered. **e**, Fab COV-2130 paratope and epitope residues involved in hydrogen bonding (dashed lines; distances in Å) and hydrophobic interactions with WA1/2020 RBD; compare with Fig. 6d showing BA.2/2130-1-0114-112 interactions. Residues forming hydrophobic interactions are shown as curved lines with rays. Atoms are shown as circles, with oxygen, carbon, and nitrogen in red, black, and blue respectively. Image created with Ligplot + . **f**, Atomic model of the RBD-Fab complex superimposed with WA1-RBD (light brown PDB: 7L7E), XBB1.1-RBD (pink PDB: 8IOS), and BQ1.1 (gray PDB 8IF2). BA.2 RBD is shown in red, with BA.2 mutations in orange. 2130-1-0114-112 HC and LC are yellow and green, with mutations in cyan and blue. Hydrogen bonds are shown as dashed lines.

To elucidate the key intermolecular interactions that form the interface and determine Omicron RBD recognition by 2130-1-0114-112, we performed 3D reconstructions of the complex between the SARS-CoV-2 Omicron BA.2 spike and the 2130-1-0114-112 Fab fragment using cryo-electron microscopy (cryo-EM). Reconstruction using refinement of the full complex gave a map with average resolution of 3.26 Å, but the interface region between the BA.2 RBD and the 2130-1-0114-112 Fab was not well resolved, presumably due to the flexibility of the RBD–Fab region in the reconstruction. To resolve details at the intermolecular interface, we performed focused refinement of this portion of the structure. Focused refinement resulted in an effective resolution of approximately 3.6 Å for this region (Electron Microscopy Data Bank EMD-28198 and EMD-28199, and Protein Data Bank 8EKD) (Fig. 6 and Extended Data Fig. 6).

This model shows the binding interface of 2130-1-0114-112–RBD and elucidates how 2130-1-0114-112 regains neutralization potency to Omicron VOCs. The parental COV2-2130 forms extensive interactions with the RBD through CDRH2 and CDRH3, as well as CDRL1 and CDRL2 (ref. ^13^) with hydrogen bond networks and hydrophobic interactions. To improve binding interactions with Omicron subvariants, 2130-1-0114-112 modifies three CDR loops: G112E in CDRH3, S32A and S33A in CDRL1, and T59E in CDRL2.

The RBD N440K substitution, identified in the DMS as sensitizing for escape from COV2-2130 but less so for 2130-1-0114-112, is on the edge of the interface with the 2130-1-0114-112 CDRH3 loop and does not make direct contact with the CDRH3 substitution G112E. However, N440K introduces a positive charge to a local environment that has substantial hydrophobic-to-hydrophobic contact. The negative charge introduced by the CDRH3 G112E substitution (Fig. 6b) might improve the electrostatic interactions in this region. It is possible that E112 and K440 are interacting by coordinating a water molecule, but the structural resolution is not sufficient to confirm this type of interaction. These experimental structural results are also consistent with our molecular dynamics simulations, which showed this transient interaction between CDRH3 E112 and RBD K440.

The local environment around the CDRL1 loop is mostly hydrophobic (comprising the RBD residues L452, F490 and L492, as well as the Omicron mutation E484A) with a hydrogen bond from LC N34 (Fig. 6c). The hydrophilic-to-hydrophobic CDRL1 substitutions introduced in 2130-1-0114-112, S32A and S33A, may favour the local environment and strengthen hydrophobic interactions with the RBD (Fig. 6c,e). This is supported by the DMS identification of sensitivity to hydrophobic-to-hydrophilic substitutions at RBD position 452 for both 1230-1-0114-112 and the parental COV2-2130. Finally, the T59E mutation in the CDRL2 loop establishes a new salt bridge with the RBD substitution Q498R present in Omicron RBDs. This new salt bridge probably strengthens the interaction with the RBD (Fig. 6d,e).

**Extended Data Fig. 7:**
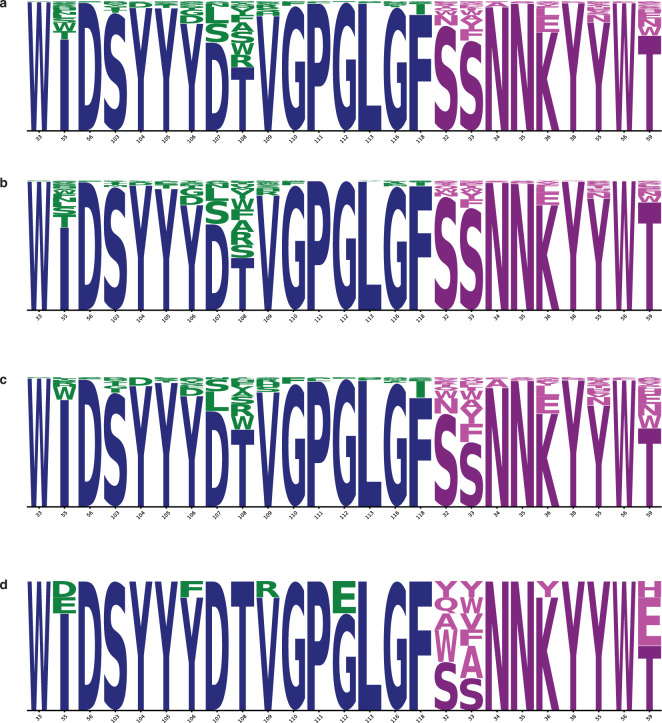
Sequence logos of candidate antibody designs. **a**, The set of 376 designed IgG includes mutation at 16 positions in the heavy chain (blue; mutations in green) and 9 positions in the light chain (magenta; mutations in pink). Height of each letter is proportional to the frequency of the amino acid in the group. **b, c**, This set of 376 sequences is divided into two overlapping sets, Set 1 (**b**; n = 230) and Set 2 (**c**; n = 204). **d**, From these two sets, a set of eight sequences was selected for production at larger scale and further evaluation including assessment of their thermostability and binding performance (Fig. 2). Selected sequences show reduction in mutations throughout the CDRH3 residues (103-118) mutated in (**a**), especially in residues 103-108.

**Extended Data Fig. 8:**
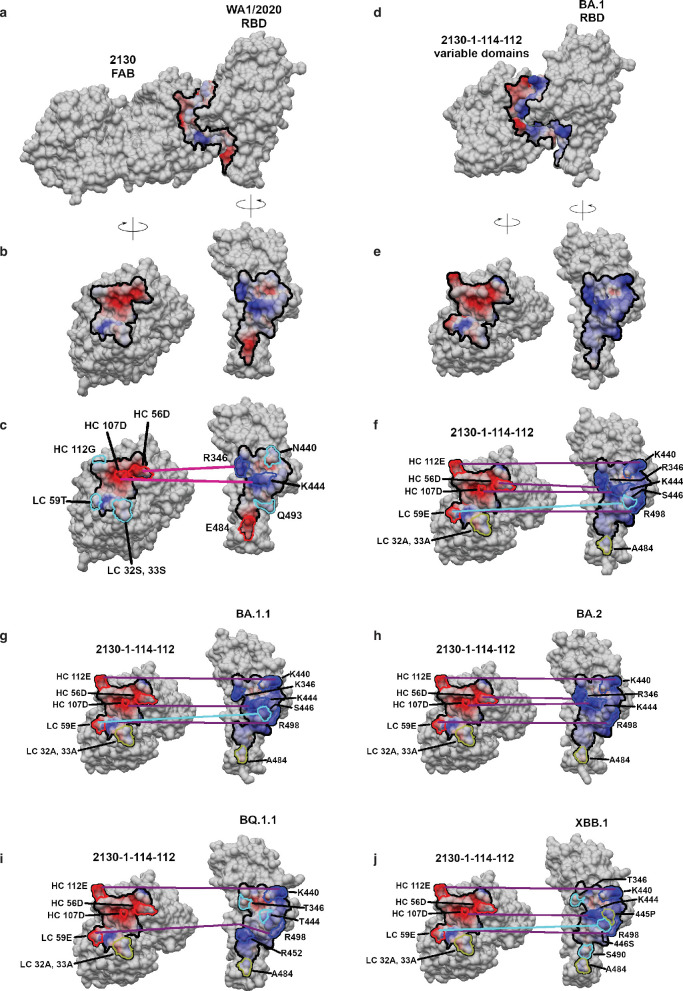
Strains with mutated epitopes are differentially neutralized by COV2-2130 and 2130-1-0114-112. **a, b**, Starting from the 7L7E structure (**a**), shown after being separated and rotated to show the contact surfaces (**b**), complexes of COV2-2130 and 2130-1-0114-112 were naively composed by rigid body superposition of the highest resolution structures available of the VOC RBDs onto the WA1/2020 RBD of 7L7E. Epitope and paratope are outlined (solid black) and colored according to charge (blue for positive, red for negative). Residues are correspondingly outlined according to charge or, if uncharged, hydrophilicity (cyan) or hydrophobicity (yellow). The models were used to infer possible intermolecular clashes and loss of key interactions that could account for loss of affinity and, conversely, relief of clashes or new favorable interactions that could account for gain of affinity. For putative interactions that differ among the antibody and RBD combinations displayed, lines show salt bridges (purple) and hydrogen bonds (cyan). **c**, The interface between COV2-2130 and WA1/2020 RBD shows a number of favorable electrostatic interactions, including between RBD R346 and HC D56 and RBD K444 and HC D107. **d-f**, In the modeled interaction between 2130-1-0114-112 and BA.1 (**d**, RBD from PDB 7X66), separated and rotated (**e**), and with interactions identified (**f**) the RBD G446S substitution changes the center of the epitope, resulting in a loss of binding with COV2-2130, perhaps by means of clashes. 2130-1-0114-112 may rescue lost affinity by introduction of LC 59E that may form a favorable interaction with R498 and S446. Also 2130-1-0114-112 may introduce a favorable interaction with all of the Omicron variants by forming a salt bridge between HC 112E and K440. Further, the designed mutations LC S32A and S33A in 2130-1-0114-112 could enhance favorable hydrophobic interaction across the interface in the A484 region. **g**, BA.1.1 RBD (RBD from PDB 7XAZ) has all of the mutations of BA.1, but additionally contains the mutation R346K, which may disrupt the favorable interaction with HC D56. **h**, BA.2 RBD (RBD from PDB 8GB8) lacks the particularly unfavorable G446S mutation present in BA.1 and BA.1.1, and the R346K mutation of BA.1.1. BA.2 also has the K440 and A484 residues that are the interaction partners of 2130-1-0114-112’s designed mutations HC E112, LC A32 and LC A33. This combination of preservation of WA1/2020 interactions and the addition of newly exploited interaction partners results in strong neutralization by 2130-1-0114-112. **i, j**, BQ.1.1 (**i**, RBD from 8IF2), and XBB.1 (**j,** RBD from 8IOS) which are not neutralized by either COV2-2130 or 2130-1-0114-112, contain substitutions in the epitope at positions 346 and either 444 or 445 as compared to WA1/2020. All three of R346, K444, and V445 are among the most highly buried residues in the epitope, and the mutation of R346 and K444 removes the two salt bridges formed with these residues. Consequently, substitutions at these positions heavily impact the shape and charge complementarity of both COV2-2130 and 2130-1-0114-112 to the RBD, likely explaining much of the impact to their affinities.

2130-1-0114-112 distributes four substitutions across three of the four CDR loops comprising the parental COV2-2130 paratope. Mutations to CDRH3 loop were less fruitful than mutations in the L1 and L2 (Extended Data Fig. 7a compared with Extended Data Fig. 7d) when looking across all antibody candidates. Among successful candidates, substitutions at positions 32 and 33 in CDRL1 appear enriched—particularly with hydrophobic residues—consistent with our analysis of this region of the experimentally solved structure of 2130-1-0114-112–BA.2 spike. Another candidate, 2130-1-0104-024, achieves improved affinity and neutralization with only two substitutions: S32W in CDRL1 and T59E in CDRL2. However, full neutralization potency is not reached without the potential charge accommodation mediated by G112E. This suggests that a combination of new bonds and accommodating charge changes optimized the restored affinity and potency of 2130-1-0114-112 with Omicron variants (Extended Data Fig. 8). Altogether, the structural model of 2130-1-0114-112 with the BA.2 RBD helps explain the observed restoration of potency to early SARS-CoV-2 Omicron VOCs.

## Discussion

We set out to rapidly design and validate derivatives of the COV2-2130 antibody that restore potent in vitro neutralization to BA.1 and BA.1.1 Omicron subvariants while maintaining binding and neutralization to previous strains of SARS-CoV-2. In addition, we sought to retain favourable thermostability properties and maintain the humanness of the sequences, a data-driven measure of similarity to known human sequences. Despite multiple mutations in the COV2-2130 epitope present in Omicron BA.1 and BA.1.1, we achieved these design objectives by applying a computationally driven, multi-objective approach. We chose to take a risk-seeking approach that increased the chance of obtaining at least one highly potent design, ideally several, by choosing diverse sequences predicted to have substantial effects on binding.

Several designed antibody candidates successfully restored neutralization potency to Omicron subvariants. In our top antibody design, 2130-1-0114-112, four substitutions accommodate Omicron escape mutations in BA.1 and BA.1.1 without sacrificing potency against Delta. This engineered antibody is thermostable; potently neutralizes Omicron BA.2, BA.4, BA.5 and BA.5.5; and restores prophylactic efficacy in vivo. Our approach for extending the utility of a high-value antibody complements state-of-the-art ex vivo discovery of high-value antibodies with responsive computational modification.

The distributed nature of the improvements in 2130-1-0114-112, with four mutations spanning three CDRs, plausibly makes this antibody comparatively robust to subsequent escape, insofar as DMS results demonstrate an improvement in binding over the parental COV2-2130 where escape substitutions present in the design targets, BA.1 and BA.1.1, are mitigated without new vulnerabilities. 2130-1-0114-112 does not mitigate the reliance of the parental COV2-2130 antibody on the RBD residue K444 and sensitivity to substitutions at this position. Although our top candidate does not neutralize the BQ.1.1 and XBB variants, which contain multiple substitutions within the COV2-2130 epitope, DMS results indicate that 2130-1-0114-112 reduces the impact of some of the mutations of the variants.

Our design approach shows potential for expediting the path of new drug products to clinical use, including lower development costs and risks versus identifying wholly new drug products of comparable breadth and efficacy. Our top-performing antibody restores in vivo efficacy and achieves potent and broad neutralization of many SARS-CoV-2 VOCs by substituting just four amino acids into a parental antibody that has been extensively tested for safety, manufacturability and clinical efficacy^1^. Given increasing evidence that neutralization is a correlate of protection from severe COVID-19 in patients treated with monoclonal antibody therapies^31,32^, an immunobridging strategy has been proposed as a response to rapidly evolving SARS-CoV-2 variants to shorten the pathway of improved monoclonal antibodies to clinical use^33^. Rapid computational rescue of high-value, potentially rare, antibodies in clinical use presents a high-impact, real-world application of our work that could be made more impactful with such an immunobridging strategy. We demonstrate successful re-targeting without requiring major sequence changes or acquisition of new liabilities. The urgency for a design approach like ours is clear given that existing antibody drug development approaches are struggling to match the rapid pace of SARS-CoV-2 evolution.

Although the individual components comprising our approach are built on existing computational approaches, we integrate them into a novel framework that demonstrates (1) a computational approach to antibody optimization that gains neutralization to a new target, (2) successful optimization of an antibody to achieve high potency to multiple targets (for example, multiple escape variants) without requiring experimental iterations, and (3) computationally restoring or improving efficacy with in vivo validation. The computational approach that we used in this work did not require iterative improvement based on feedback from experimental evaluations, nor did it require availability of data on antibody candidates tested against the target antigens, either of which would result in further delays when responding to an emergent variant. Furthermore, our fundamental approach is adaptable to more modest or decentralized computing resources than those used in this study.

Future work seeks to extend our computational approach to include additional predictive models, such as models predicting antibody expression, protein aggregation and polyreactivity. Our models for predicting antibody–antigen binding heavily depend on performing simulations with sufficiently accurate models of antibody–antigen co-structures, which is an important limitation. Consequently, we are developing experimental datasets to advance machine learning-based approaches for predicting binding directly from sequence, as well as incorporating emerging artificial intelligence-based approaches for determining and refining structural models.

In this study, we demonstrate an innovative computational methodology capable of creating an array of antibody designs targeting the initial subvariants of Omicron SARS-CoV-2. A subset of these designed antibodies display enhancements over the parental COV2-2130 antibody, including superior binding, broad and potent neutralization, and in vivo protection against Omicron BA.1.1. Our approach demonstrates an adaptable antibody-based therapeutic discovery strategy, enabling rapid deployment in response to emerging viral threats or evolutionary shifts. Furthermore, the limited number of amino acid substitutions in our redesigned antibodies suggests the feasibility of an immunobridging strategy for accelerated regulatory approval, especially if the parental antibody has received regulatory clearance for use in humans. Our computational method can also proactively mitigate liabilities identified via DMS, potentially limiting the impact of escape variants and thereby extending the therapeutic utility of the designed antibody in a clinical setting.

### Reporting summary

Further information on research design is available in the Nature Portfolio Reporting Summary linked to this article.

## Online content

Any methods, additional references, Nature Portfolio reporting summaries, source data, extended data, supplementary information, acknowledgements, peer review information; details of author contributions and competing interests; and statements of data and code availability are available at 10.1038/s41586-024-07385-1.

### Supplementary information

Supplementary Information This file contains Supplementary Methods; Supplementary Figs 1–2; Supplementary Tables 1–8

Reporting Summary

### Source data

Source Data Fig. 4

## Figures and Tables

**Extended Data Table 1:**
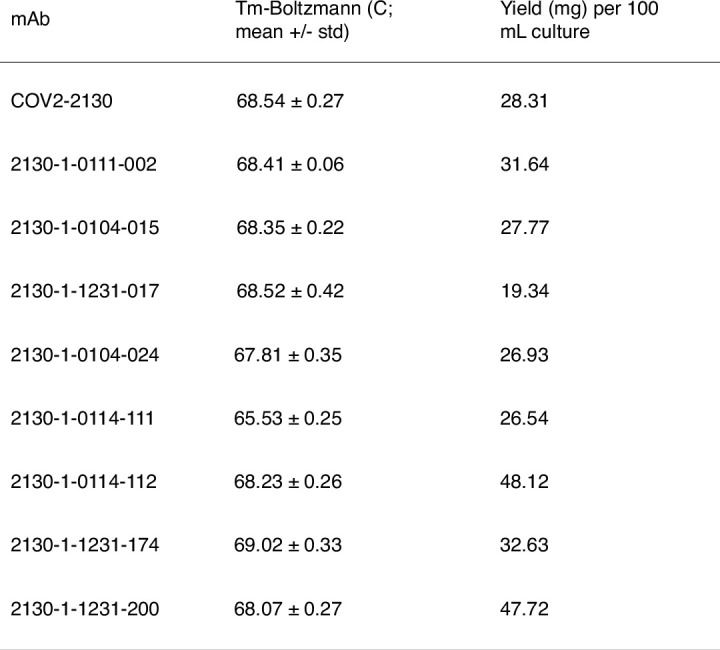
Thermal stability and expression yield of selected IgGs

Melting temperature (Tm) was determined using a fluorescence-based protein thermal shift assay (GloMelt^TM^, Biotium). Yield was determined by measuring optical density at 280 nm and deriving antibody quantity using the calculated extinction coefficient.

**Extended Data Table 2:**
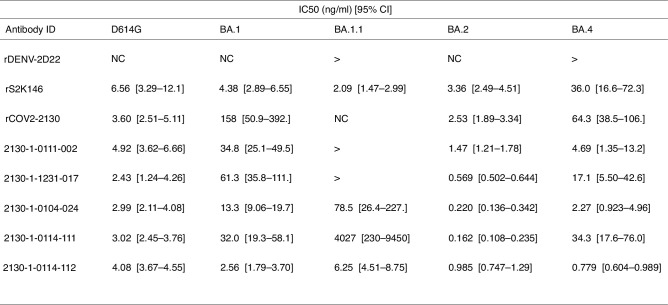
Pseudoviral neutralization potency (IC50) of selected IgGs, corresponding to Fig. 3

Pseudovirus neutralization IC50 values and 95% confidence intervals corresponding to Fig. 3. “>” indicates a value > 10,000 ng/mL; NC indicates positive hill slope or failure to converge.

## Data Availability

The EM map and model have been deposited to the Electron Microscopy Data Bank (EMD-28198 and EMD-28199) and the Protein Data Bank (PDB; 8EKD). Other protein structural data were used in this work (PDB 7L7E and 7T9K) and in analysis (PDB 7X66, 7XAZ, 8IOS, 8IF2 and 8GB8). Sequence data that support the findings of this study have been deposited in GenBank under accessions PP474664–PP474679 and are available in the Supplementary Information. Source data for Fig. 4 are provided with the paper. DMS library variant data and antibody per replicate DMS selection data can be accessed at https://github.com/dms-vep/SARS-CoV-2_Omicron_BA.2_spike_DMS_COV2-2130 and https://github.com/dms-vep/SARS-CoV-2_Omicron_BA.1_spike_DMS_COV2-2130 GitHub repositories. Source data are provided with this paper.

## Code availability

The ImprovWF dynamic workflows code is available at https://github.com/LLNL/improvwf. Selected GUIDE workflow components are available at https://github.com/LLNL/CRPCA. All DMS data analysis was performed using dms-vep-pipeline version 1.8, which can be accessed at https://github.com/dms-vep/dms-vep-pipeline/tree/51e73d601bd770eb6e9abd21f57fb4365699c984. Code and notebooks related to DMS runs are available at https://dms-vep.github.io/SARS-CoV-2_Omicron_BA.1_spike_DMS_COV2-2130/ and https://dms-vep.github.io/SARS-CoV-2_Omicron_BA.2_spike_DMS_COV2-2130/.
