# Supplementary material for: Computationally restoring the potency of a clinical antibody against Omicron
Source: Nature. 2024 May 8;629(8013):878–85. doi: 10.1038/s41586-024-07385-1 (PMC11111397; doi:10.1038/s41586-024-07385-1)
Supplement: Supplementary file 1 — This file contains Supplementary Methods; Supplementary Figs 1–2; Supplementary Tables 1–8 [file 41586_2024_7385_MOESM1_ESM.pdf]

nature portfolio

https://doi.org/10.1038/s41586-024-07385-1

Supplementary information

Computationally restoring the potency of a clinical antibody against Omicron

In the format provided by the authors and unedited

nature portfolio

https://doi.org/10.1038/s41586-024-07385-1

Supplementary information

Computationally restoring the potency of a clinical antibody against Omicron

In the format provided by the authors and unedited

Supplementary Methods

Problem formulation: Generating antibody-antigen co-structures. To best manage the high sensitivity of protein binding affinity (herein considered as mutational changes to the binding free energy, ddG) predictions to antibody–antigen structure quality[35], we used the program LGA[36] to evaluate compatibility between numerous experimentally solved structures of the receptor binding domains (RBD), available structures of the Fab form of COV2-2130, and structures of RBD-Fab complexes. This approach allowed us to identify regions of backbone and side-chain deviation (see **Fig. SM1a**).

We used the conformational centroid to select a representative complex for further analysis. Structural clustering of tested RBDs identified Omicron RBD (PDB id 7t9k, chain A) as the centroid of all evaluated conformations (shown on **Fig. SM1b**). We consequently chose to perform ddG calculations on two initial structures (**Fig. SM1c**): an experimentally solved structure of WT RBD with the Fab form of COV2-2130 (PDB ID 7l7e, chains S, M, N), and a structural model of Omicron RBD complexed with COV2-2130 (PDB ID 7l7e, chains M, N) that uses the RBD as the identified conformational centroid (PDB ID 7t9k chain A).

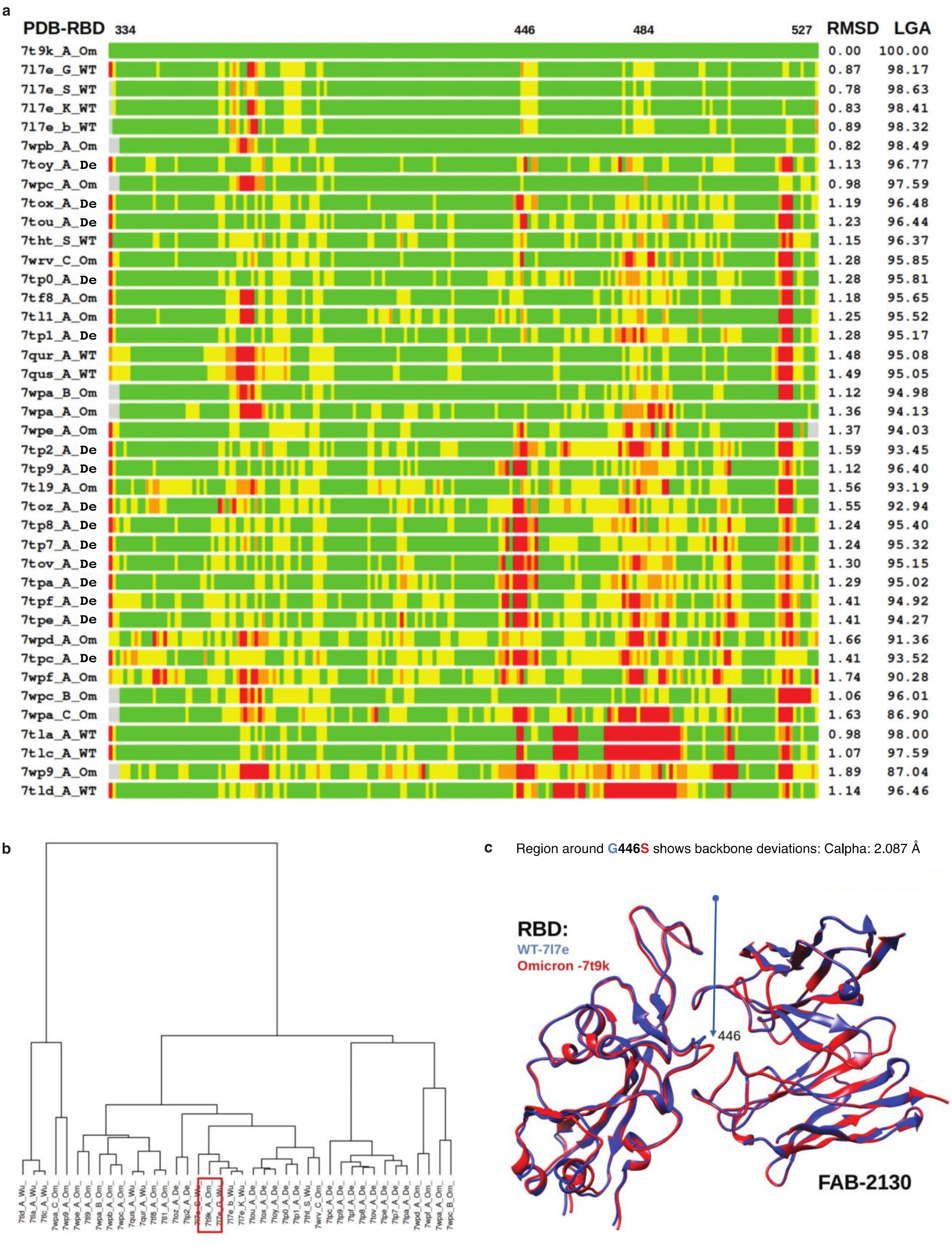

Supplementary Methods Fig. 1 | Bar plot representation of superpositions of the selected

reference Omicron RBD structure (PDB ID 7t9k chain A) with 40 RBDs from WT, Omicron or Delta variants and comparison of experimentally solved RBD structures.

a, The RMSD (Å) and LGA structure similarity scores (0-100) were calculated against the reference structure are provided in the right columns. Deviations of < 1Å are shown in green, 1-2 Å in yellow, 2-4 Å in orange, and > 4Å in red. The regions in RBD within the RBD-Fab interface where the major structural deviations between Omicron, Delta and WT are observed (positions 446 and 484) are marked at the top. **b**, Structural clustering by LGA of 40 experimentally-solved RBDs. A red rectangle marks an identified centroid (an RBD from Omicron PDB ID 7t9k chain A) **c**, A structure of WT RBD-COV2-2130 (PDB ID 7L7E, blue) superimposed with a model of Omicron RBD-COV2-2130 (Derived from PDB ID 7T9K, red). A significant deviation between two models is observed in the RBD-Fab interface in the region surrounding a mutation position WT to Omicron G446S (arrow).

Problem formulation: Defining the search space. We specify which antibody positions to consider for mutation based on our estimated co-structures. We consider a given position for mutation if its wild-type residue includes any atoms less than 7 Å from any antigen atom. Under this criterion, we consider 25 positions for mutation. For each position considered, we allow amino acid substitutions to all amino acids except cysteine or proline. We limit each mutant sequence to a maximum of 9 amino acid substitutions relative to wild-type COV2-2130. This results in a search space of size $\sum_{k=1}^{9} \binom{25}{k} \cdot 17^k \approx 2.5 \times 10^{17}$.

Semi-autonomous system. In the two sections below, we describe how our semi-autonomous system proposes mutant antibody sequences and selects which sequences to simulate via Rosetta Flex[18]. Decision-making agents run in parallel in an asynchronous, distributed fashion. Each agent operates on a single HPC node and selects a number of sequences to simulate according to the number of cores on that node. Simulation results (e.g., Rosetta Flex ddG calculations) are recorded in a centralized database so all agents have access to all results. Initially, the system simulates all single-point mutant sequences using MD, SFE, FEP, Rosetta Flex, and FoldX, as well as scores them under AbBERT.

Sequence generation. Mutant antibody sequences are proposed using a sequence generator that operates using a hierarchical sampling process. First, the number of mutations is uniform randomly sampled from 1 to 8. Then, mutations are sampled without replacement according to a probability distribution informed by six tools: Atomistic MD, SFE, FEP, Rosetta Flex, FoldX, and AbBERT. Concretely, each tool outputs a score (e.g., -ddG for MD) for all single-point mutant

sequences. Each score is then converted into an unnormalized mutation probability by passing it through a generalized logistic function:

$$l(\text{score}) = \frac{1}{(1 + a \cdot e^{-\text{score}\cdot b})^{1/c}},$$

where $a = 1000$, $b = 5$, and $c = 2$ were hand-tuned to strongly prefer positive scores and to effectively squash or truncate extreme values (e.g., ddG's of -3 and -3.5 produce very similar scores). Finally, we obtain a normalized probability distribution by normalizing across all tools and all possible mutations. Thus, the probability of mutation m is given by:

$$p(m) = \frac{\sum_{i=1}^{N_t} l\big(\text{score}_i(m)\big)}{\sum_{i=1}^{N_t}\sum_{j=1}^{N_m} l\left(\text{score}_i\big(m_j\big)\right)},$$

where N_t is the number of tools, N_m is the number of possible mutations, score_i is the score of the ith tool, and m_j is the jth possible mutation.

Sequence selection. We employ Bayesian optimization agents to select which sequences to simulate via Rosetta Flex. Within the optimization loop, we first generate batches of sequences from our sequence generator. We then use Gaussian process (GP) agents, described below, to estimate the posterior distribution for each proposed sequence and select a subset of sequences according to the maximum expected improvement (MEI) acquisition function[37]. Finally, the subset of sequences are simulated using Rosetta Flex, and the optimization loop continues.

The input to the GP is based on a count-based featurization of the mutant antibody sequence and a multilayer perceptron (MLP). The count-based featurization is based on chemical and size

properties of mutant sequences, described as follows. In the starting, unmutated co-structure, we identify pairs of antibody-antigen amino acids with α-carbon to α-carbon distances less than 10 Å. This establishes a bipartite graph of antibody and antigen amino acids, where the vertices are antibody and antigen amino acids and the edges are the identified antibody-antigen amino acid pairs. Given a mutant sequence, we substitute amino acid mutations at the vertices without altering the graph structure. For each vertex (amino acid), we assign one or more chemical properties (acidic, aliphatic, aromatic, basic, hydroxylic, sulfuric); see **Table ST1**. For each edge (antibody amino acid-antigen amino acid), according to the two connected vertices, we assign one or more unordered pairs of chemical properties (e.g., acidic-sulfuric, basic-basic), of which there are 28 possible pairs. Similarly, we assign each vertex a size property (very small, small, medium, large, very large; see **Table ST1**) and each edge an unordered pair of size properties (e.g., small-medium), of which there are 15 possible pairs. We define the first 43 features as the counts of each chemical and size property pair. The second 43 features are these same counts when using wild-type antibody.

The resulting 86-dimensional feature vector is then used as input to a multilayer perceptron (MLP), comprising a single hidden layer with output dimension 40 and tanh activation, followed by an output layer with output dimension 10 and no activation. The output of the MLP is then used as the input to the GP. The MLP and GP are implemented in PyTorch[39] and GPyTorch[40], respectively.

The GP is defined by a mean and kernel covariance function $GP(\mu(x), k(x, x'))$, a non-zero constant prior mean function $\mu(x) = C$, where C is a learned scalar parameter, and a scaled radial basis function kernel operating on inputs x_1 and x_2:

$$k(x_1, x_2) = \lambda \cdot \exp\left(-\frac{1}{2}(x_1 - x_2)^{\mathrm{T}}\Theta^{-2}(x_1 - x_2)\right),$$

where Θ is a length scale parameter and λ is a kernel scale parameter. GP parameters (C, Θ, λ) and MLP parameters were jointly trained using stochastic gradient descent on the log marginal likelihood of Rosetta Flex ddG values of 20,000 training sequences from the sequence generator with respect to the GP likelihood function, $p(y \mid X)$.

We compute the GP predictive posterior distribution f_* as $p(f_* \mid X_*, X, y)$, where X is the union of the 20,000 training sequences and 10,000 sequences randomly drawn from the centralized database (all passed through the featurization and MLP), and y represents the union of the corresponding Rosetta Flex ddG values. To select a batch of sequences, we first generate 1,000 candidate sequences, X_*, from the sequence generator. We compute marginal posteriors for each sequence, then select the sequence with the maximum expected improvement, where we are minimizing the free energy:

$$\mathrm{EI}(x) = \big(f(x') - \mu(x)\big)\Phi\left(\frac{f(x') - \mu(x)}{\sigma(x)}\right) + \sigma(x)\phi\left(\frac{f(x') - \mu(x)}{\sigma(x)}\right),$$

where x is the candidate sequence, x' is the sequence with lowest ddG acquired so far, μ(x) is the mean of the GP predictive posterior at x, $\sigma(x)$ is the standard deviation of the GP posterior at x, and Φ and ϕ are the cumulative density and probability density functions of the normal distribution. Upon selection, X and y are updated using the selected sequence and the

predicted ddG, respectively. Then the predictive posterior f_* is re-computed. Subsequently, we select the next candidate according to MEI; this process continues until enough sequences are selected to occupy all cores.

To supplement the sequences chosen by the Bayesian optimization agents, and to ensure sufficient coverage in sequence space, we employ two additional types of rules-based agents. The first agent simply selects all combinations of two-point mutant sequences. The second agent samples from among the current top-performing sequences (i.e., those with the most negative ddG) and further mutates them according to the sequence generator described above.

Down-selecting sequences for experimental validation. The autonomous system described above produced over 125,000 sequences simulated using Rosetta Flex. To down-select this set to our experimental capacity of 376 candidate sequences, we first computed the Pareto (non-dominated) set of sequences, based on the objectives listed in **Table ST2**. Note that MD, SFE, and FEP multi-point mutation scores were approximated as the sum of their constituent single-point mutation scores; multi-point AbBERT scores were computed on all sequences. The resulting Pareto set contained 3,809 sequences. From here, we sought consensus across tools by ranking all sequences in the Pareto set according to the weighted sum of each objective, with a penalty based on mutational distance from wild-type COV2-2130:

$$f(m) = -0.5 \cdot d^2(m, m_0) + \sum_i w_i \cdot g_i(m),$$

where m is a mutant sequence, m_0 is wild-type COV2-2130, g_i is the ith objective, w_i is the ith weight, and d is the count of amino acid substitutions relative to wild-type COV2-2130. We set

these weights appropriate to both the relative importance and the scales of the individual predictors.

We expect that some simulations will systematically misestimate the value of some mutations. Enforcing sequence diversity of selected antibodies may mitigate risk of systematic errors in our simulation tools. Thus, we enforced sequence diversity when selecting among the top-ranked sequences. First, we limited the number of times any particular mutation could appear in the final set; lower-ranked sequences beyond this limit were excluded from selection. Second, for each tool, we enforced inclusion of at least one sequence containing that tool's top-performing single-point mutations among the set considered, even if these were disfavored by other tools. Third, to ensure mutational diversity across positions, we enforced inclusion of at least one sequence containing a mutation at interface positions, even if not scored favorably by the tools. Finally, we excluded sequences containing more than four mutations to aromatic residues and sequences containing glycosylation motifs. After eliminating enforced exclusions from the ranked list and selecting enforced inclusions, we selected remaining top-ranked sequences.

Atomistic molecular dynamics (MD) simulations for free energies as affinity predictions. We performed MD simulations by implementing workflows using the publicly available program OpenMM (v7.4)[41] with CHARMM36 parameters[42]. Complexes were first solvated in an isotropic TIP3P[43] box. K+ and Cl- ions were then added to neutrality and 150 mM concentration. After energy minimization, we ran MD simulations with a Langevin integrator (1 ps-1)[44]. Monte Carlo barostat (303.15 K), particle mesh ewald summation (1 Å grid)[45], and SHAKE[46]. Simulations proceeded in 2 fs timesteps for a total of 125 ps with constraints on backbone and side chain atoms (400 and 40 kJ/mol·nm2, respectively). An additional 10 ns were then run without constraints.

From the final coordinates, we increased sampling using a minimum watershell[47] with adaptive boundary and hydrogen masses were increased to 4 amu by transferring the mass from the bonded non-hydrogen atom. These simulations employed a 4 fs time step[48], 300K thermostat, and particle mesh ewald electrostatics. The antibody and antigen were separated by 8 Å, with individual simulations under harmonic constraints (100 kcal/mol·Å^2) at 1 Å intervals. At each 1 Å interval, we ran 4 ns of re-equilibration and an additional 320 ns of MD to provide sampling needed to calculate the free energy[49]. Sampling of the CDR loops might benefit from using a force field tuned to reproduce conformations of intrinsically unstructured proteins[50].

Structural Fluctuation Estimation (SFE) approach for reproducible and robust free energy prediction. We applied our Structural Fluctuation Estimation (SFE) approach[17] to address problems of reproducibility and robustness of calculated estimates in energy changes upon

mutations (ddG). Antibody-antigen structures were minimized and relaxed using standard minimization procedures from Rosetta[51], Chimera[52], and GROMACS[53] steepest descent and conjugate gradient methods; short MD simulations in GROMACS subsequently extracted a set of structure snapshots from the resulting trajectories.

For each initial structure, we generated 60 structural conformations for the RBD-Fab complex—30 complexes with mutations, and 30 without. Each set of 30 complexes includes the initial structure, 4 minimized structures, and 25 structures from MD trajectories, so as to capture structural uncertainties, possible structural deviations upon introduced mutations, and natural fluctuations in protein structure. Using the established Rosetta Flex ddG protocol[18], we performed mutational ddG calculations in the "forward" direction on models without mutations, and in "reverse" on models with mutations. Once ddG calculations were completed, we removed outliers, averaged results of the interquartile simulations, and calculated the final ddG estimate via the formula: $ddG = (ddG_{forward} – ddG_{reverse}) / 2$. The resulting ddG value provides an affinity estimate shown to be more reproducible and robust than ddG estimates calculated from only one initial input structure of the RBD-Fab complex, whether using standard FoldX[19], Rosetta[54], or Flex ddG[18] procedures.

Free energy perturbation calculations. Free energy perturbation (FEP) is an established, rigorous, physics-based method for calculating free energy differences that employs MD simulations. As reported recently[20], we implemented an automated protocol for large-scale FEP calculations to evaluate the effect of antibody mutation on conformational stability. The

structure of the COV2-2130 Fab was taken from the crystal structure 7L7E. Using the FEP protocol described[20], we calculated the change in antibody conformational stability for all single-point mutations. We first considered an extended set of 29 residues to assess whether COV2-2130 exhibited stability liabilities outside the 25 residues described above. We subsequently limited mutations to only the 25 positions in the multi-point optimization.

AbBERT language model. AbBERT[21] is a transformer-based language model derived by fine-tuning the pre-trained ProtBERT[55] language model on over 200,000 human antibody sequences obtained from the Observed Antibody Space (OAS) database[22]. The trained AbBERT model estimates the distribution of human antibody sequences, providing a way to measure the resemblance of candidate antibodies to human antibodies. We scored the humanness of mutant sequences via a multi-unmask scoring procedure.

Antigen production. To express the RBD subdomain of the SARS-CoV-2 S protein, residues 328–531 were cloned into a mammalian expression vector downstream of a mu-phosphatase signal peptide and upstream of an AviTag and a 8×His tag. Three previously identified stabilizing mutations (Y365F, F392W, V395I) were included in the RBD to enhance antigen stability and yield. For RBD constructs corresponding to the Omicron subvariants, mutations present in each subvariant were introduced into the context of the stabilized, wild-type RBD construct. RBD constructs were transfected into Expi293F cells (ThermoFisher Scientific), and expressed protein was isolated by metal affinity chromatography on HisTrap Excel columns (Cytiva). This cell line tested negative for mycoplasma in regular testing. For structural studies, we used a

previously described stabilized SARS-CoV-2 spike construct (VFLIP). This construct contains an inter-protomer disulfide bond, a shortened linker between the S1 and S2 domains, and five proline substitutions relative to the native sequence of SARS-CoV-2 spike. In addition to these modifications, this construct also contains a c-terminal T4 fibritin foldon domain as well as an 8×His tag and a TwinStrep tag for purification. To express the protein, we transfected Expi293F cells (ThermoFisher Scientific) with a plasmid encoding SARS-CoV-2 S_VFLIP with BA.2 amino acid substitutions. Culture supernatants were collected 4-5 d after transfection and clarified by centrifugation. BioLock (IBA Biosciences) was added to remove free biotin in the culture media, after which supernatants were filter-sterilized using a 0.2 µm filter. Full-length VFLIP_BA.2 was purified by streptactin affinity chromatography using StrepTrap HP columns (Cytiva) and eluted using an elution buffer of 25 mM desthiobiotin in Dulbecco's phosphate-buffered saline (DPBS). After elution, spike protein was prepared for use in cryo-electron microscopy (Cryo-EM) by size-exclusion chromatography. Purified proteins were analyzed by SDS-PAGE to assess purity and appropriate molecular weights.

Antibody production. For each antibody in the first set of 230 designs, nucleotide sequences encoding the designed heavy and light chain sequences were synthesized, cloned into an hIgG1 framework, and used to produce mAbs via transient transfection of HEK293 cells at ATUM (Newark, CA, USA). This cell line tested negative for mycoplasma.

For the second set of 204 designs, monoclonal antibody sequences were synthesized (Twist Bioscience), cloned into an IgG1 monocistronic expression vector[56] (designated as pVVC-mCisK_hG1), and expressed either at microscale in transiently transfected ExpiCHO cells[57] for screening, or at larger scale for down-stream assays. This cell line tested negative for mycoplasma in regular testing. Sequences in this group of 204 designs all contain an additional arginine at the beginning of the light chain constant region with respect to sequences expressed in the first set. Larger-scale monoclonal antibody expression was performed by transfecting (30 ml per antibody) CHO cell cultures using the Gibco ExpiCHO Expression System and protocol for 125ml flasks (Corning) as described by the vendor. Culture supernatants were purified using HiTrap MabSelect SuRe (Cytiva, formerly GE Healthcare Life Sciences) on a 24-column parallel protein chromatography system (Protein BioSolutions). Purified monoclonal antibodies were buffer-exchanged into PBS and stored at 4 °C until use. Amino acid and DNA sequences of the top 8 antibodies that were selected for production at larger scale and further evaluation are provided in **Tables ST3-7**.

Binding screening and characterization. Immunoassays for screening the first set of 230 designs (**Fig. ED1**) and later characterization were performed on the Gyrolab xPlore instrument (Gyros Protein Technologies) using the Bioaffy 200 discs (Gyros Protein Technologies). The standard manufacturer's immunoassay automated protocol was executed with fluorescence detection set to 0.1% PMT. Assay column washes were performed in PBS + 0.02% Tween 20 (PBST). Capture antigens were applied to the assay columns at 0.5 to 2.0 µM in PBS. Analyte mAbs were applied to the assay columns diluted in PBST at 1:200 for single-concentration

screening or as a serial dilution from 1,000 nM to 0.25 nM for characterization of down-selected candidate antibodies. A secondary detection antibody served as a fluorescent reporter: Alexa Fluor 647 AffiniPure Fab Fragment Goat Anti-Human IgG, Fcγ fragment specific (Jackson ImmunoResearch) diluted to 50-100 nM in RexxipF buffer (Gyros Protein Technologies). Resulting values were fit to a 4PL model or calculated as area under the curve (AUC) using GraphPad Prism software.

Dose-response ELISA binding assays. For screening and characterizing the second set of 204 designs (**Fig. ED2**), wells of 384-well microtiter plates were coated with purified recombinant SARS-CoV-2 RBD proteins at 4 °C overnight at an antigen concentration of 2 mg/mL. Plates were washed with Dulbecco's phosphate-buffered saline (DPBS) containing 0.05% Tween-20 (DPBS-T) and blocked with 2% bovine serum albumin and 2% normal goat serum in DPBS-T (blocking buffer) for 1 h. mAbs were diluted in 12 three-fold serial dilutions in blocking buffer at a starting concentration of 10 µg/mL. Plates were then washed and mAb dilutions were added and incubated for 1 h. Plates were washed, a goat anti-human IgG conjugated with horseradish peroxidase (HRP) (Southern Biotech, cat. 2014-05, lot L2118-VG00B, 1:5,000 dilution in blocking buffer) was added, and the plates were incubated for 1 h. After plates were washed, signal was developed with a 3,3',5,5'-tetramethylbenzidine (TMB) substrate (Thermo Fisher Scientific). Color development was monitored, 1M hydrochloric acid was added to stop the reaction, and the absorbance was measured at 450 nm using a spectrophotometer (Biotek). Dose-response ELISAs were performed in technical triplicate with at least two independent experimental replicates.

Thermal Shift Protein Assays (melt-curve assays). Antibody concentrations were determined using the Qubit Protein Assay Kit (ThermoFisher). The GloMelt™ Thermal Shift Protein Stability Kit (Biotum) was utilized to determine the thermal stability of the antibodies by following the manufacturer's suggested protocols. The analysis was performed using a melt-curve program on an ABI 7500 Fast Dx Real-Time PCR instrument. Each assay was done in triplicate, using 5ug of mAb per well. The raw melt curve data was imported into and analyzed via Protein Thermal Shift ™ software version 1.4 (ThermoFisher) to generate the melting temperature and fit data.

Pseudovirus Neutralization. Pseudovirus neutralization assays were carried out according to the protocol of Crawford *et al.*[58] One day prior to the assay, 293T cells stably expressing human ACE2 (293T-hACE2 cells) were seeded onto 96-well tissue culture plates coated with poly-D-lysine. The day of the assay, serial dilutions of monoclonal antibodies in duplicate were prepared in a 96-well microtiter plate and pre-incubated with pseudovirus for 1 h at 37 °C in the presence of a final concentration of 5 mg/mL polybrene (EMD Millipore), before the pseudovirus-mAb mixtures were added to 293T-hACE2 monolayers. Plates were returned to the 37 °C incubator, and then 48-60 h later luciferase activity was measured on a CLARIOStar plate reader (BMG LabTech) using the Bright-Glo Luciferase Assay System (Promega). Percent inhibition of pseudovirus infection was calculated relative to pseudovirus-only control.
IC50 values were determined by nonlinear regression using Prism v.8.1.0 (GraphPad). Each neutralization assay was repeated at least twice.

Viruses: FRNT and in vivo protection. The WA1/2020 recombinant strain with D614G substitution and B.1.617.2 was described previously[28,59]. The BA.1 isolate was obtained from an individual in Wisconsin as a mid-turbinate nasal swab[60]. The BA.1.1 and BA.2 strains were obtained from nasopharyngeal isolates. The BA.2.12.1, BA.4, BA.5, and BA.5.5 isolates were generous gifts from M. Suthar (Emory University), A. Pekosz (Johns Hopkins University), and R. Webby (St. Jude Children's Research Hospital). All viruses were passaged once on Vero-TMPRSS2 cells and subjected to next-generation sequencing[61] to confirm the introduction and stability of substitutions. All virus experiments were performed in an approved biosafety level 3 (BSL-3) facility.

Focus Reduction Neutralization Test. Serial dilutions of sera were incubated with 10^2 focus-forming units (FFU) of WA1/2020 D614G, B.1.617.2, BA.1, BA.1.1, BA.2, BA.2.12.1, BA.4, BA.5, or BA.5.5 for 1 h at 37°C. Antibody-virus complexes were added to Vero-TMPRSS2 cell monolayers in 96-well plates and incubated at 37°C for 1 h. Subsequently, cells were overlaid with 1% (w/v) methylcellulose in MEM. Plates were harvested 30 h (WA1/2020 D614G and B.1.617.2) or 70 h (BA.1, BA.1.1, BA.2, BA.2.12.1, BA.4, BA.5, and BA.5.5) later by removing overlays and fixed with 4% PFA in PBS for 20 min at room temperature. Plates were washed and sequentially incubated with a pool (SARS2-02, -08, -09, -10, -11, -13, -14, -17, -20, -26, -27, -28, -31, -38, -41, -42, -44, -49, -57, -62, -64, -65, -67, and -71)[62] of anti-S murine antibodies (including cross-reactive mAbs to SARS-CoV) and HRP-conjugated goat anti-mouse IgG (Sigma Cat # A8924, RRID: AB_258426) in PBS supplemented with 0.1% saponin and 0.1% bovine serum albumin. SARS-CoV-2-infected cell foci were visualized using TrueBlue peroxidase substrate

(KPL) and quantitated on an ImmunoSpot microanalyzer (Cellular Technologies). The pool of SARS-CoV-2 antibodies is not commercially available.

Mouse studies. Animal studies were carried out in accordance with the recommendations in the Guide for the Care and Use of Laboratory Animals of the National Institutes of Health. The protocols were approved by the Institutional Animal Care and Use Committee at the Washington University School of Medicine (assurance number A3381–01). Virus inoculations were performed under anesthesia that was induced and maintained with ketamine hydrochloride and xylazine, and all efforts were made to minimize animal suffering.

No sample sizes were chosen a priori but instead estimated based on prior knowledge of anticipated experimental differences among groups. All experiments with statistical analysis were repeated at least two independent times, each with multiple technical replicates. Experimental size of animal cohorts was determined based on prior experience performing studies in mice. No data was excluded. All experiments had multiple biological and/or technical replicates and are indicated the Figure legend. For animal studies, mice were randomly assigned from large batches obtained from the vendor to different experimental groups in an age-matched distribution. No blinding was performed as handling of BSL3 virus requires exact tracking of infected mice and samples.

Seven to nine-week-old female heterozygous K18-hACE2 C57BL/6J mice (strain: 2B6.Cg-Tg(K18-ACE2)2Prlmn/J, Cat # 34860) were obtained from The Jackson Laboratory and used. Mice were

housed in groups of 3 to 5. Photoperiod = 12 hr on:12 hr off dark/light cycle. Ambient animal room temperature is 70° F, controlled within ±2° and room humidity is 50%, controlled within ±5%. No wild animals were used in this study. No field collected samples were used in this study. Animals were housed in groups and fed standard chow diets.

Mice were administered 100 µg of 2130-1-0114-112, parental 2130, or isotype control anti-West Nile virus hE16 mAb[63] by intraperitoneal injection one day before intranasal inoculation with 104 focus-forming units (FFU) of WA1/2020 D614G, BA.1.1 or BA.5. Animals were euthanized at 4 days post-infection and tissues were harvested for virological analysis.

Measurement of Viral RNA burden. Tissues were weighed and homogenized with zirconia beads in a MagNA Lyser instrument (Roche Life Science) in 1 ml of DMEM medium supplemented with 2% heat-inactivated FBS. Tissue homogenates were clarified by centrifugation at 10,000 rpm for 5 min and stored at −80°C. RNA was extracted using the MagMax mirVana Total RNA isolation kit (Thermo Fisher Scientific) on the Kingfisher Flex extraction robot (Thermo Fisher Scientific). RNA was reverse transcribed and amplified using the TaqMan RNA-to-CT 1-Step Kit (Thermo Fisher Scientific). Reverse transcription was carried out at 48°C for 15 min, followed by 2 min at 95°C. Amplification was accomplished over 50 cycles as follows: 95°C for 15 s and 60°C for 1 min. Copies of SARS-CoV-2 *N* gene RNA in samples were determined using a published assay[64].

Plaque Assay Neutralization Tests. All SARS-CoV 2 viral stocks and VAT cells used for plaque assays were obtained through BEI Resources, NIAID, NIH. Delta variant (isolate hCoV-19/USA/MD-HP05647/2021, lineage B.1.617.2; NR-55672) was contributed by Dr. Andrew S. Pekosz. BA.1 (isolate hCoV-19/USA/GA-EHC-2811C/2021, lineage B.1.1.529; NR-56481) was contributed by Mehul Suthar. BA1.1 (isolate hCoV-19/USA/HI-CDC-4359259-001/2021, lineage B.1.1.529; NR-56475) was contributed by Centers for Disease Control. Viral stocks were amplified in Vero E6 cells (Delta variant) or VAT cells (Omicron variants). Serial dilutions of mAbs were incubated with virus at a concentration of 400 PFU/mL at 37°C with 5% CO2 for 30 min. Antibody-virus complexes were then added to VAT cells in 12-well plates and incubated for 30 min, then overlaid with 2 mL per well of 0.6% microcrystalline cellulose (Sigma) in minimal essential media (ThermoFisher) supplemented with 0.3% bovine serum albumin (Sigma), and 1% penicillin/streptomycin (ThermoFisher). After 72 hours incubation, plaques were visualized by incubation in 0.25% crystal violet in 100% methanol for 10 minutes. All virus experiments were performed in an approved biosafety level 3 (BSL-3) facility.

Deep Mutational Scanning. BA.1 full spike deep mutational scanning libraries were designed as described previously[29]. BA.2 full spike deep mutational scanning libraries were designed using the same methods as BA.1 libraries except using BA.2 spike as a template sequence. The sequence of BA.2 spike can be found at https://github.com/dms-vep/SARS-CoV-2_Omicron_BA.2_spike_DMS_COV2-2130/blob/main/library_design/reference_sequences/3332_pH2rU3_ForInd_Omicron_sinobiological_BA2_B11529_Spiked21_T7_CMV_ZsGT2APurR.gb. For antibody escape mapping

experiments, each library was incubated for 1 h at 37˚C with increasing amounts of COV2-2130 or 2130-1-0114-112 antibodies. For COV2-2130, starting antibody concentration for the BA.1 libraries was 50 µg/ml and increased 4- and 8-fold; for the BA.2 libraries, the starting concentration was 0.32 µg/ml and increased 5- and 25-fold. For 2130-1-0114-112, starting antibody concentration for the BA.1 libraries was 0.16 µg/ml; for the BA.2 library, starting concentration was 0.11 µg/ml and in both cases concentrations were increased 5 and 25-fold. After incubation virus-antibody mix was used to infect HEK-293T-ACE2 cells[58] and viral genomes were recovered for deep sequencing 12 h after infection. Two biological replicates (virus libraries with independent sets of mutations) were used for each antibody mapping.

Escape for each mutation in the library was quantified using a non-neutralized control as described previously[29]. This analysis uses a biophysical model described previously[65] and implemented in the polyclonal package found at https://jbloomlab.github.io/polyclonal/. Full analysis pipeline for each antibody can be found at https://dms-vep.org/SARS-CoV-2_Omicron_BA.1_spike_DMS_COV2-2130/ for BA.1 libraries and at https://dms-vep.org/SARS-CoV-2_Omicron_BA.2_spike_DMS_COV2-2130/ for BA.2 libraries.

Cryo-EM sample preparation and data collection. The Fab 2130-1-0114-112 and Omicron BA.2 were expressed recombinantly and combined in a molar ration of 1:4 (Ag:Fab). The mixture was incubated overnight at 4°C and purified by gel filtration. 2.2µl of the purified mixture at a concentration of 0.5 mg/mL was applied to glow discharged (30 s at 25mA) grid (300 mesh 1.2/1.3, Quantifoil). The grids were blotted for 3.5 s before plunging into liquid ethane using

Vitrobot MK4 (TFS) at 20°C and 100% RH. Grids were screened on a Glacios (TFS) microscope and imaged on Krios operated at 300 keV equipped with a K3 and GIF (Gatan) DED detector using counting mode. Movies were collected at nominal magnification of 130,000X, pixel size of 0.647 Å/pixel and defocus range of 0.8 to 1.8 µm. Grids were exposed at ~1.09 $e^-/Å^2$/frame resulting in total dose of ~52.2 $e^-/Å^2$ (**Table ST8**).

Cryo-EM data processing. Data processing was performed with Relion 4.0 beta2[66]. Movies were preprocessed with Relion Motioncor2[67] and CTFFind4[68]. Micrographs with low resolution, high astigmatism, and defocus were removed from the data set. The data set was first manually picked to generate 2D images and then autopicked by Relion template picker[69] and subject to 2D and 3D classification. Good classes were used for another round of autopicking with Topaz training and Topaz picking[66,70]. The particles were extracted in a box size of 600 pixel and binned to 96 pixels (pixel size of 4.04 Å/pixel). The particles were subjected to multiple rounds of 2D class averages, 3D initial map and 3D classification without symmetry to obtain a clean homogeneous particle set. This set was re-extracted at a pixel size of 1.516 Å/pixel and was subjected to 3D autorefinement. The data were further re-extracted at a pixel size of 1.29Å/pixel and processed with CTFrefine, polished[71] and subjected to final 3D autorefinement and postprocessing resulting in ~3.26Å map. To better resolve the area of interaction between Cov2-RBD/2130-1-0114-112, a focused refinement was performed by particles expansion (C3 symmetry) and signal subtraction with masking around the RBD/2130-1-0114-112. The subtracted particles were subjected to 3D classification without alignment and selected

particles were subjected to 3D autorefinement and postprocessing resulting in ~3.7Å map.

Detailed statistics are provided in **Fig. SM2** and **Table ST8**.

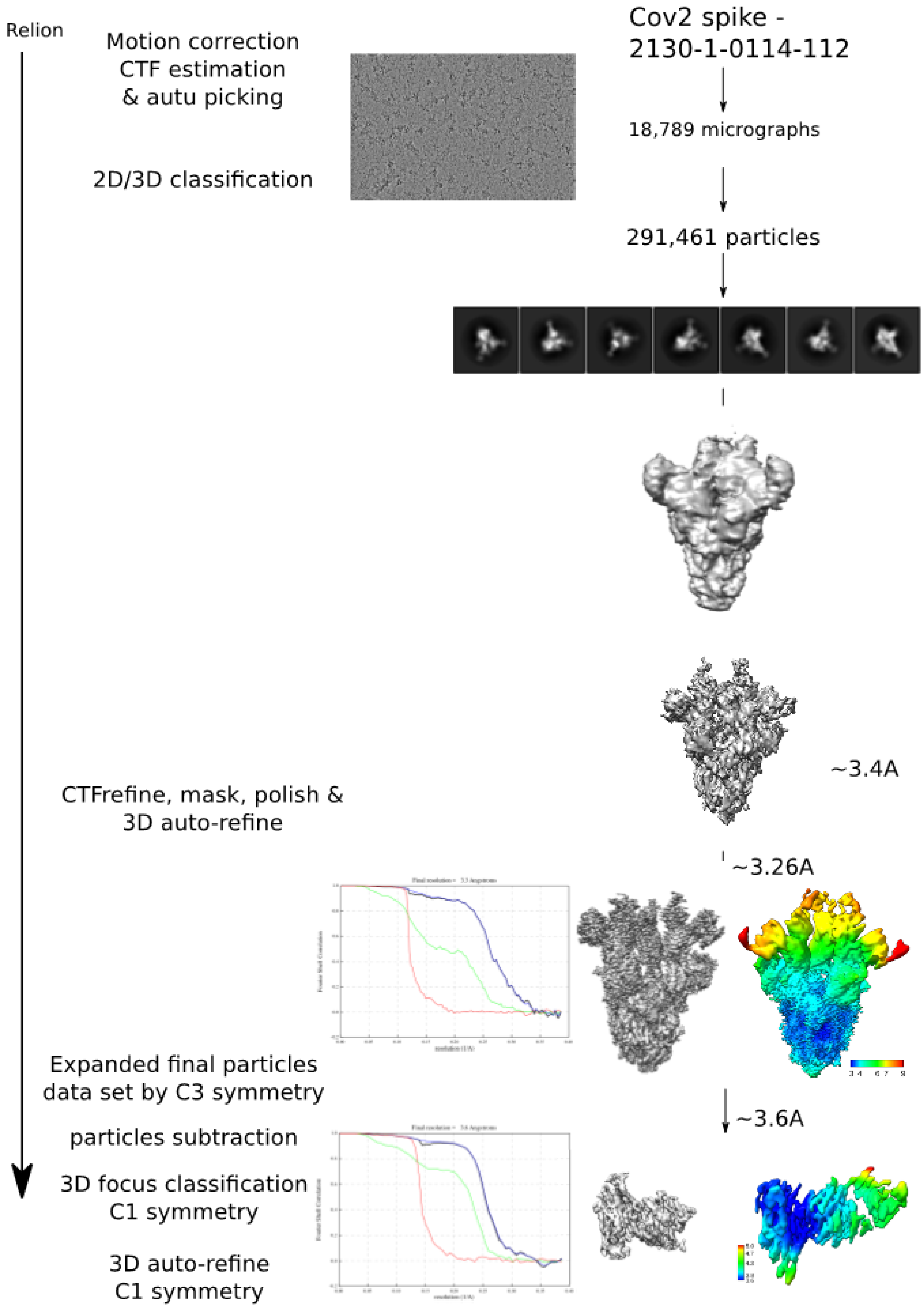
Relion
Motion correction
CTF estimation
& autu picking
2D/3D classification
Cov2 spike -
2130-1-0114-112
18,789 micrographs
291,461 particles
~3.4A
CTFrefine, mask, polish &
3D auto-refine
~3.26A
Expanded final particles
data set by C3 symmetry
~3.6A
particles subtraction
3D focus classification
C1 symmetry
3D auto-refine
C1 symmetry

Supplementary Methods Fig. 2 | CryoEM workflow of SARS-CoV-2 BA.2 spike bound to Fab. Workflow proceeds temporally from top to bottom including collection, picking, classification, the generation of an initial, full-spike complex, and focus refinement conducted at the Fab/RBD. At the bottom, Gold-standard Fourier shell correlation curves and maps are colored by local resolution calculated using Relion, before and after local refinement.

Model building and refinement. For model building PDB: 7L7E[13] was used for initial modelling of the RBD and the 2130-1-0114-112 Fv. All models were first docked to the map with Chimera[52] or ChimeraX[72]. To improve coordinates, the models were subjected to iterative refinement of manual building in Coot[73] and Phenix[74,75]. The models were validated with Molprobity[76] (**Table ST8**). The EM map and model has been deposited into EMDB (EMD-28198, EMD-28199) and PDB (8EKD).

References:

35 Geng, C., Xue, L. C., Roel-Touris, J. & Bonvin, A. M. J. J. Finding the ΔΔ *G* spot: Are predictors of binding affinity changes upon mutations in protein–protein interactions ready for it? *WIREs Computational Molecular Science* **9**, doi:10.1002/wcms.1410 (2019).

36 Zemla, A. LGA: a method for finding 3D similarities in protein structures. *Nucleic Acids Research* **31**, 3370–3374, doi:10.1093/nar/gkg571 (2003).

37 Mockus, J. The application of Bayesian methods for seeking the extremum. *Towards global optimization* **2**, 117 (1998).

38 Pommié, C., Levadoux, S., Sabatier, R., Lefranc, G. & Lefranc, M.-P. IMGT standardized criteria for statistical analysis of immunoglobulin V-REGION amino acid properties. *Journal of Molecular Recognition* **17**, 17-32, doi:https://doi.org/10.1002/jmr.647 (2004).

39 Gardner, J., Pleiss, G., Weinberger, K. Q., Bindel, D. & Wilson, A. G. Gpytorch: Blackbox matrix-matrix gaussian process inference with gpu acceleration. *Advances in neural information processing systems* **31** (2018).

40 Paszke, A. *et al.* Pytorch: An imperative style, high-performance deep learning library. *Advances in neural information processing systems* **32** (2019).

41 Eastman, P. *et al.* OpenMM 7: Rapid development of high performance algorithms for molecular dynamics. *PLOS Computational Biology* **13**, e1005659, doi:10.1371/journal.pcbi.1005659 (2017).

42 Huang, J. & MacKerell, A. D. CHARMM36 all-atom additive protein force field: Validation based on comparison to NMR data. *Journal of Computational Chemistry* **34**, 2135–2145, doi:10.1002/jcc.23354 (2013).

43 Jorgensen, W. L., Chandrasekhar, J., Madura, J. D., Impey, R. W. & Klein, M. L. Comparison of simple potential functions for simulating liquid water. *The Journal of Chemical Physics* **79**, 926-935 (1983).

44 Salomon-Ferrer, R., Götz, A. W., Poole, D., Le Grand, S. & Walker, R. C. Routine Microsecond Molecular Dynamics Simulations with AMBER on GPUs. 2. Explicit Solvent

Particle Mesh Ewald. *Journal of Chemical Theory and Computation* **9**, 3878–3888, doi:10.1021/ct400314y (2013).

45 Darden, T., York, D. & Pedersen, L. Particle mesh Ewald: An *N* ·log(*N*) method for Ewald sums in large systems. *The Journal of Chemical Physics* **98**, 10089–10092, doi:10.1063/1.464397 (1993).

46 Ryckaert, J.-P., Ciccotti, G. & Berendsen, H. J. C. Numerical integration of the cartesian equations of motion of a system with constraints: molecular dynamics of n-alkanes. *Journal of Computational Physics* **23**, 327–341, doi:10.1016/0021-9991(77)90098-5 (1977).

47 Ovchinnikov, V., Conti, S., Lau, E. Y., Lightstone, F. C. & Karplus, M. Microsecond Molecular Dynamics Simulations of Proteins Using a Quasi-Equilibrium Solvation Shell Model. *Journal of Chemical Theory and Computation* **16**, 1866–1881, doi:10.1021/acs.jctc.9b01072 (2020).

48 Hopkins, C. W., Le Grand, S., Walker, R. C. & Roitberg, A. E. Long-Time-Step Molecular Dynamics through Hydrogen Mass Repartitioning. *Journal of Chemical Theory and Computation* **11**, 1864–1874, doi:10.1021/ct5010406 (2015).

49 Ovchinnikov, V., Nam, K. & Karplus, M. A Simple and Accurate Method To Calculate Free Energy Profiles and Reaction Rates from Restrained Molecular Simulations of Diffusive Processes. *The Journal of Physical Chemistry B* **120**, 8457–8472, doi:10.1021/acs.jpcb.6b02139 (2016).

50 Huang, J. *et al.* CHARMM36m: an improved force field for folded and intrinsically disordered proteins. *Nature methods* **14**, 71-73 (2017).

51 Rohl, C. A., Strauss, C. E. M., Chivian, D. & Baker, D. Modeling structurally variable regions in homologous proteins with rosetta. *Proteins: Structure, Function, and Bioinformatics* **55**, 656–677, doi:10.1002/prot.10629 (2004).

52 Pettersen, E. F. *et al.* UCSF Chimera—A visualization system for exploratory research and analysis. *Journal of Computational Chemistry* **25**, 1605–1612, doi:10.1002/jcc.20084 (2004).

53 Abraham, M. J. *et al.* GROMACS: High performance molecular simulations through multi-level parallelism from laptops to supercomputers. *SoftwareX* **1-2**, 19–25, doi:10.1016/j.softx.2015.06.001 (2015).

54 Kortemme, T. & Baker, D. A simple physical model for binding energy hot spots in protein–protein complexes. *Proceedings of the National Academy of Sciences* **99**, 14116–14121, doi:10.1073/pnas.202485799 (2002).

55 Elnaggar, A. *et al.* ProtTrans: Towards Cracking the Language of Lifes Code Through Self-Supervised Deep Learning and High Performance Computing. *IEEE Transactions on Pattern Analysis and Machine Intelligence*, 1–1, doi:10.1109/TPAMI.2021.3095381 (2021).

56 Chng, J. *et al.* Cleavage efficient 2A peptides for high level monoclonal antibody expression in CHO cells. *mAbs* **7**, 403–412, doi:10.1080/19420862.2015.1008351 (2015).

57 Zost, S. J. *et al.* Rapid isolation and profiling of a diverse panel of human monoclonal antibodies targeting the SARS-CoV-2 spike protein. *Nature Medicine* **26**, 1422–1427, doi:10.1038/s41591-020-0998-x (2020).

58 Crawford, K. H. D. *et al.* Protocol and Reagents for Pseudotyping Lentiviral Particles with SARS-CoV-2 Spike Protein for Neutralization Assays. *Viruses* **12**, 513, doi:10.3390/v12050513 (2020).

59 Plante, J. A. *et al.* Spike mutation D614G alters SARS-CoV-2 fitness. *Nature* **592**, 116–121, doi:10.1038/s41586-020-2895-3 (2021).

60 Halfmann, P. J. *et al.* SARS-CoV-2 Omicron virus causes attenuated disease in mice and hamsters. *Nature* **603**, 687–692, doi:10.1038/s41586-022-04441-6 (2022).

61 Chen, R. E. *et al.* Resistance of SARS-CoV-2 variants to neutralization by monoclonal and serum-derived polyclonal antibodies. *Nature Medicine* **27**, 717–726, doi:10.1038/s41591-021-01294-w (2021).

62 VanBlargan, L. A. *et al.* A potently neutralizing SARS-CoV-2 antibody inhibits variants of concern by utilizing unique binding residues in a highly conserved epitope. *Immunity* **54**, 2399–2416.e2396, doi:10.1016/j.immuni.2021.08.016 (2021).

63 Oliphant, T. *et al.* Development of a humanized monoclonal antibody with therapeutic potential against West Nile virus. *Nature Medicine* **11**, 522–530, doi:10.1038/nm1240 (2005).

64 Case, J. B., Bailey, A. L., Kim, A. S., Chen, R. E. & Diamond, M. S. Growth, detection, quantification, and inactivation of SARS-CoV-2. *Virology* **548**, 39–48, doi:10.1016/j.virol.2020.05.015 (2020).

65 Yu, T. C. *et al.* A biophysical model of viral escape from polyclonal antibodies. *Virus Evolution* **8** (2022).

66 Kimanius, D., Dong, L., Sharov, G., Nakane, T. & Scheres, S. H. W. New tools for automated cryo-EM single-particle analysis in RELION-4.0. *Biochemical Journal* **478**, 4169–4185, doi:10.1042/BCJ20210708 (2021).

67 Zheng, S. Q. *et al.* MotionCor2: anisotropic correction of beam-induced motion for improved cryo-electron microscopy. *Nature Methods* **14**, 331–332, doi:10.1038/nmeth.4193 (2017).

68 Rohou, A. & Grigorieff, N. CTFFIND4: Fast and accurate defocus estimation from electron micrographs. *Journal of Structural Biology* **192**, 216–221, doi:10.1016/j.jsb.2015.08.008 (2015).

69 Fernandez-Leiro, R. & Scheres, S. H. W. A pipeline approach to single-particle processing in *RELION*. *Acta Crystallographica Section D Structural Biology* **73**, 496–502, doi:10.1107/S2059798316019276 (2017).

70 Bepler, T., Kelley, K., Noble, A. J. & Berger, B. Topaz-Denoise: general deep denoising models for cryoEM and cryoET. *Nature Communications* **11**, 5208, doi:10.1038/s41467-020-18952-1 (2020).

71 Zivanov, J. *et al.* New tools for automated high-resolution cryo-EM structure determination in RELION-3. *eLife* **7**, e42166, doi:10.7554/eLife.42166 (2018).

72 Pettersen, E. F. *et al.* UCSF ChimeraX: Structure visualization for researchers, educators, and developers. *Protein Science* **30**, 70-82 (2021).

73 Emsley, P. & Cowtan, K. *Coot* : model-building tools for molecular graphics. *Acta Crystallographica Section D Biological Crystallography* **60**, 2126–2132, doi:10.1107/S0907444904019158 (2004).

74 Adams, P. D. *et al. PHENIX* : a comprehensive Python-based system for macromolecular structure solution. *Acta Crystallographica Section D Biological Crystallography* **66**, 213–221, doi:10.1107/S0907444909052925 (2010).

75 Afonine, P. V. *et al.* Real-space refinement in *PHENIX* for cryo-EM and crystallography. *Acta Crystallographica Section D Structural Biology* **74**, 531–544, doi:10.1107/S2059798318006551 (2018).

76 Chen, V. B. *et al. MolProbity* : all-atom structure validation for macromolecular crystallography. *Acta Crystallographica Section D Biological Crystallography* **66**, 12–21, doi:10.1107/S0907444909042073 (2010).

Supplementary Tables

Supplementary Table 1. | Amino acid chemical and size classifications used in the count-based featurization; modified from Pommié, 2004[38].

Size\Chemical Class	Aliphatic	Aromatic	Acidic	Basic	Hydroxilic	Sulfuric	Amidic
Very Large		F, W, Y					
Large	I, L			K, R		M	
Medium	V		E	H			Q
Small	P		D		T	C	N
Very Small	A, G				S		

Supplementary Table 2| Objectives considered for Pareto selection of candidate sequences, with associated units and weights.

Objective (g_i)	Units	Weight (w_i)
Rosetta Flex ddG, L452R (Delta)	Rosetta Energy Units	-1.0
Rosetta Flex ddG, BA.1	REU	-1.0
Rosetta Flex ddG, BA.1.1	REU	-1.0
Atomistic MD ddG BA.1	KCal/Mol	-1.5
SFE ddG, BA.1	REU	-1.5
SFE ddG, BA.1.1	REU	-1.5
FoldX ddG, L452R (Delta)	KCal/Mol	-0.5
FoldX ddG, BA.1	KCal/Mol	-0.5
FoldX ddG, BA.1.1	KCal/Mol	-0.5
FEP stability ddG	KCal/Mol	-0.5
AbBERT score	Arbitrary Units (AU)	2.0

Supplementary Table 3 | Selected sequence records as designed (Corresponding to Fig. ED7d):

>md5_e3d1904966eaf73aed30331f60b658e9 Mutant_number: md5_e3d1904966eaf73aed30331f60b658e9 HumID: 2130-1-1231-017
Master: COV2-2130 Mutations: IH55E,YH106F,SL32W,SL33A
EVQLVESGGGLVKPGGSLRLSCAASGFTFRDVWMSWVRQAPGKGLEWVGRIKSKEDGGTTDYAAPVKGRFTISRDDSKNTLYLQMNSLKTEDTAV
YYCTTAGSYYFDTVGPGLPEGKFDYWGQGTLVTVSS
DIVMTQSPDSLAVSLGERATINCKSSQSVLYWANNKNYLAWYQQKPGQPPKLLMYWASTRESGVPDRFSGSGSGAEFTLTISSLQAEDVAIYYCQ
QYYSTLTFGGGTKVEIKR
>md5_d323d72bb6bc337ea256bdd92ceba08e Mutant_number: md5_d323d72bb6bc337ea256bdd92ceba08e HumID: 2130-1-1231-174
Master: COV2-2130 Mutations: VH109R
EVQLVESGGGLVKPGGSLRLSCAASGFTFRDVWMSWVRQAPGKGLEWVGRIKSKIDGGTTDYAAPVKGRFTISRDDSKNTLYLQMNSLKTEDTAV
YYCTTAGSYYYDTRGPGLPEGKFDYWGQGTLVTVSS
DIVMTQSPDSLAVSLGERATINCKSSQSVLYSSNNKNYLAWYQQKPGQPPKLLMYWASTRESGVPDRFSGSGSGAEFTLTISSLQAEDVAIYYCQ
QYYSTLTFGGGTKVEIKR
>md5_aaca5fa01a0f27e83e39da01a4457347 Mutant_number: md5_aaca5fa01a0f27e83e39da01a4457347 HumID: 2130-1-1231-200
Master: COV2-2130 Mutations: SL33W
EVQLVESGGGLVKPGGSLRLSCAASGFTFRDVWMSWVRQAPGKGLEWVGRIKSKIDGGTTDYAAPVKGRFTISRDDSKNTLYLQMNSLKTEDTAV
YYCTTAGSYYYDTVGPGLPEGKFDYWGQGTLVTVSS
DIVMTQSPDSLAVSLGERATINCKSSQSVLYSWNNKNYLAWYQQKPGQPPKLLMYWASTRESGVPDRFSGSGSGAEFTLTISSLQAEDVAIYYCQ
QYYSTLTFGGGTKVEIKR
>md5_689536fbc67d8ee33e80ca5a6709d167 Mutant_number: md5_689536fbc67d8ee33e80ca5a6709d167 HumID: 2130-1-0111-002
Master: COV2-2130 Mutations: IH55D,SL33F,TL59E
EVQLVESGGGLVKPGGSLRLSCAASGFTFRDVWMSWVRQAPGKGLEWVGRIKSKDDGGTTDYAAPVKGRFTISRDDSKNTLYLQMNSLKTEDTAV
YYCTTAGSYYYDTVGPGLPEGKFDYWGQGTLVTVSS
DIVMTQSPDSLAVSLGERATINCKSSQSVLYSFNNKNYLAWYQQKPGQPPKLLMYWASERESGVPDRFSGSGSGAEFTLTISSLQAEDVAIYYCQ
QYYSTLTFGGGTKVEIKR
>md5_a314041f0f349a3dc6b474c069163d9b Mutant_number: md5_a314041f0f349a3dc6b474c069163d9b HumID: 2130-1-0114-111
Master: COV2-2130 Mutations: GH112E,SL32Y,SL33V,KL36Y
EVQLVESGGGLVKPGGSLRLSCAASGFTFRDVWMSWVRQAPGKGLEWVGRIKSKIDGGTTDYAAPVKGRFTISRDDSKNTLYLQMNSLKTEDTAV
YYCTTAGSYYYDTVGPELPEGKFDYWGQGTLVTVSS
DIVMTQSPDSLAVSLGERATINCKSSQSVLYYVNNYNYLAWYQQKPGQPPKLLMYWASTRESGVPDRFSGSGSGAEFTLTISSLQAEDVAIYYCQ
QYYSTLTFGGGTKVEIKR
>md5_3052c528d578179c00211647380e5fb7 Mutant_number: md5_3052c528d578179c00211647380e5fb7 HumID: 2130-1-0114-112
Master: COV2-2130 Mutations: GH112E,SL32A,SL33A,TL59E
EVQLVESGGGLVKPGGSLRLSCAASGFTFRDVWMSWVRQAPGKGLEWVGRIKSKIDGGTTDYAAPVKGRFTISRDDSKNTLYLQMNSLKTEDTAV
YYCTTAGSYYYDTVGPELPEGKFDYWGQGTLVTVSS
DIVMTQSPDSLAVSLGERATINCKSSQSVLYAANNKNYLAWYQQKPGQPPKLLMYWASERESGVPDRFSGSGSGAEFTLTISSLQAEDVAIYYCQ
QYYSTLTFGGGTKVEIKR
>md5_2041aa110908c8cde47ed7aabc074be8 Mutant_number: md5_2041aa110908c8cde47ed7aabc074be8 HumID: 2130-1-0104-015
Master: COV2-2130 Mutations: SL32Q,SL33Y,TL59H
EVQLVESGGGLVKPGGSLRLSCAASGFTFRDVWMSWVRQAPGKGLEWVGRIKSKIDGGTTDYAAPVKGRFTISRDDSKNTLYLQMNSLKTEDTAV
YYCTTAGSYYYDTVGPGLPEGKFDYWGQGTLVTVSS
DIVMTQSPDSLAVSLGERATINCKSSQSVLYQYNNKNYLAWYQQKPGQPPKLLMYWASHRESGVPDRFSGSGSGAEFTLTISSLQAEDVAIYYCQ
QYYSTLTFGGGTKVEIKR
>md5_1089bd14be7c511aa67dbef73eeb1e42 Mutant_number: md5_1089bd14be7c511aa67dbef73eeb1e42 HumID: 2130-1-0104-024
Master: COV2-2130 Mutations: SL32W,TL59E
EVQLVESGGGLVKPGGSLRLSCAASGFTFRDVWMSWVRQAPGKGLEWVGRIKSKIDGGTTDYAAPVKGRFTISRDDSKNTLYLQMNSLKTEDTAV
YYCTTAGSYYYDTVGPGLPEGKFDYWGQGTLVTVSS
DIVMTQSPDSLAVSLGERATINCKSSQSVLYWSNNKNYLAWYQQKPGQPPKLLMYWASERESGVPDRFSGSGSGAEFTLTISSLQAEDVAIYYCQ
QYYSTLTFGGGTKVEIKR

Supplementary Table 4 | Selected sequence records as constructed: IgG heavy-chain DNA sequences.

```
>2130-1-1231-017_heavy_chain Redesigned COV2-2130 candidate 2130-1-1231-017 heavy chain
GAAGTGCAGCTCGTGGAGTCGGGTGGCGGACTTGTGAAGCCCGGGGGATCACTGCGGTTGTCCTGTGCCGCCTCGGGTTTTACCTTCCGCGACGT
GTGGATGAGCTGGGTCAGACAGGCGCCGGGAAAGGGACTGGAATGGGTCGGCAGGATCAAGTCCAAAGAGGACGGGGGCACCACCGATTACGCAG
CCCCAGTGAAGGGCCGCTTCACCATTTCACGGGACGACTCCAAGAACACCCTGTATCTGCAAATGAACTCCCTCAAGACTGAAGATACGGCCGTG
TACTACTGCACAACCGCTGGCAGCTACTACTTCGACACTGTGGGACCGGGACTGCCTGAGGGAAAGTTCGATTACTGGGGCCAGGGTACCCTCGT
GACTGTCAGCTCCGCCAGCACCAAGGGCCCATCGGTCTTCCCCCTGGCACCCTCCTCCAAGAGCACCTCTGGGGGCACAGCGGCCCTGGGCTGCC
TGGTCAAGGACTACTTCCCCGAACCGGTGACGGTGTCGTGGAACTCAGGCGCCCTGACCAGCGGCGTGCACACCTTCCCGGCTGTCCTACAGTCC
TCAGGACTCTACTCCCTCAGCAGCGTGGTGACCGTGCCCTCCAGCAGCTTGGGCACCCAGACCTACATCTGCAACGTGAATCACAAGCCCAGCAA
CACCAAGGTGGACAAGAAAGTTGAGCCCAAATCTTGTGACAAAACTCACACATGCCCACCGTGCCCAGCACCTGAACTCCTGGGGGGACCGTCAG
TCTTCCTCTTCCCCCCAAAACCCAAGGACACCCTCATGATCTCCCGGACCCCTGAGGTCACATGCGTGGTGGTGGACGTGAGCCACGAAGACCCT
GAGGTCAAGTTCAACTGGTACGTGGACGGCGTGGAGGTGCATAATGCCAAGACAAAGCCGCGGGAGGAGCAGTACAACAGCACGTACCGGGTGGT
CAGCGTCCTCACCGTCCTGCACCAGGACTGGCTGAATGGCAAGGAGTACAAGTGCAAGGTCAGCAACAAAGCCCTCCCAGCCCCCATCGAGAAAA
CCATCTCCAAAGCCAAAGGGCAGCCCCGAGAACCACAGGTGTACACCCTGCCCCCATCCCGGGATGAGCTGACCAAGAACCAGGTCAGCCTGACC
TGCCTGGTCAAAGGCTTCTATCCCAGCGACATCGCCGTGGAGTGGGAGAGCAATGGGCAGCCGGAGAACAACTACAAGACCACGCCTCCCGTGCT
GGACTCCGACGGCTCCTTCTTCCTCTACAGCAAGCTCACCGTGGACAAGAGCAGGTGGCAGCAGGGGAACGTCTTCTCATGCTCCGTGATGCATG
AGGCTCTGCACAACCACTACACGCAGAAGTCCCTCTCCCTGTCTCCGGGTAAATAG
>2130-1-1231-174_heavy_chain Redesigned COV2-2130 candidate 2130-1-1231-174 heavy chain
GAAGTGCAGCTCGTGGAATCCGGGGGCGGACTCGTGAAGCCCGGGGGAAGCCTGCGGCTGTCGTGCGCGGCTTCAGGGTTCACCTTCCGCGACGT
GTGGATGTCCTGGGTCAGACAGGCCCCAGGAAAGGGACTGGAATGGGTCGGCAGGATTAAGTCCAAGATCGACGGTGGCACCACCGATTACGCAG
CCCCTGTGAAGGGCCGGTTCACCATCTCCCGGGACGATTCCAAGAACACGCTGTACTTGCAAATGAACAGCCTGAAAACTGAGGACACCGCCGTG
TACTACTGTACTACCGCCGGCTCCTACTACTATGATACTCGCGGTCCGGGACTTCCCGAGGGAAAGTTTGACTACTGGGGACAGGGCACCCTCGT
GACTGTGTCGAGCGCCAGCACCAAGGGCCCATCGGTCTTCCCCCTGGCACCCTCCTCCAAGAGCACCTCTGGGGGCACAGCGGCCCTGGGCTGCC
TGGTCAAGGACTACTTCCCCGAACCGGTGACGGTGTCGTGGAACTCAGGCGCCCTGACCAGCGGCGTGCACACCTTCCCGGCTGTCCTACAGTCC
TCAGGACTCTACTCCCTCAGCAGCGTGGTGACCGTGCCCTCCAGCAGCTTGGGCACCCAGACCTACATCTGCAACGTGAATCACAAGCCCAGCAA
CACCAAGGTGGACAAGAAAGTTGAGCCCAAATCTTGTGACAAAACTCACACATGCCCACCGTGCCCAGCACCTGAACTCCTGGGGGGACCGTCAG
TCTTCCTCTTCCCCCCAAAACCCAAGGACACCCTCATGATCTCCCGGACCCCTGAGGTCACATGCGTGGTGGTGGACGTGAGCCACGAAGACCCT
GAGGTCAAGTTCAACTGGTACGTGGACGGCGTGGAGGTGCATAATGCCAAGACAAAGCCGCGGGAGGAGCAGTACAACAGCACGTACCGGGTGGT
CAGCGTCCTCACCGTCCTGCACCAGGACTGGCTGAATGGCAAGGAGTACAAGTGCAAGGTCAGCAACAAAGCCCTCCCAGCCCCCATCGAGAAAA
CCATCTCCAAAGCCAAAGGGCAGCCCCGAGAACCACAGGTGTACACCCTGCCCCCATCCCGGGATGAGCTGACCAAGAACCAGGTCAGCCTGACC
TGCCTGGTCAAAGGCTTCTATCCCAGCGACATCGCCGTGGAGTGGGAGAGCAATGGGCAGCCGGAGAACAACTACAAGACCACGCCTCCCGTGCT
GGACTCCGACGGCTCCTTCTTCCTCTACAGCAAGCTCACCGTGGACAAGAGCAGGTGGCAGCAGGGGAACGTCTTCTCATGCTCCGTGATGCATG
AGGCTCTGCACAACCACTACACGCAGAAGTCCCTCTCCCTGTCTCCGGGTAAATAG
>2130-1-1231-200_heavy_chain Redesigned COV2-2130 candidate 2130-1-1231-200 heavy chain
GAAGTGCAACTGGTGGAGAGCGGCGGGGGCCTTGTGAAGCCCGGCGGCTCCCTGCGCTTGTCATGCGCGGCCTCCGGTTTTACCTTCCGGGACGT
GTGGATGAGCTGGGTCAGACAGGCCCCTGGAAAAGGGCTGGAATGGGTCGGACGCATCAAGTCCAAGATCGACGGGGGAACGACAGACTACGCAG
CTCCAGTGAAGGGCCGGTTCACCATTAGCCGGGACGACTCGAAGAACACCCTGTATCTCCAAATGAACTCCCTCAAGACCGAAGATACCGCCGTG
TACTACTGCACTACCGCCGGTTCCTACTACTACGATACTGTGGGCCCGGGACTGCCCGAGGGAAAGTTCGATTACTGGGGACAGGGAACCCTGGT
CACTGTGTCGTCCGCCAGCACCAAGGGCCCATCGGTCTTCCCCCTGGCACCCTCCTCCAAGAGCACCTCTGGGGGCACAGCGGCCCTGGGCTGCC
TGGTCAAGGACTACTTCCCCGAACCGGTGACGGTGTCGTGGAACTCAGGCGCCCTGACCAGCGGCGTGCACACCTTCCCGGCTGTCCTACAGTCC
TCAGGACTCTACTCCCTCAGCAGCGTGGTGACCGTGCCCTCCAGCAGCTTGGGCACCCAGACCTACATCTGCAACGTGAATCACAAGCCCAGCAA
CACCAAGGTGGACAAGAAAGTTGAGCCCAAATCTTGTGACAAAACTCACACATGCCCACCGTGCCCAGCACCTGAACTCCTGGGGGGACCGTCAG
TCTTCCTCTTCCCCCCAAAACCCAAGGACACCCTCATGATCTCCCGGACCCCTGAGGTCACATGCGTGGTGGTGGACGTGAGCCACGAAGACCCT
GAGGTCAAGTTCAACTGGTACGTGGACGGCGTGGAGGTGCATAATGCCAAGACAAAGCCGCGGGAGGAGCAGTACAACAGCACGTACCGGGTGGT
CAGCGTCCTCACCGTCCTGCACCAGGACTGGCTGAATGGCAAGGAGTACAAGTGCAAGGTCAGCAACAAAGCCCTCCCAGCCCCCATCGAGAAAA
CCATCTCCAAAGCCAAAGGGCAGCCCCGAGAACCACAGGTGTACACCCTGCCCCCATCCCGGGATGAGCTGACCAAGAACCAGGTCAGCCTGACC
TGCCTGGTCAAAGGCTTCTATCCCAGCGACATCGCCGTGGAGTGGGAGAGCAATGGGCAGCCGGAGAACAACTACAAGACCACGCCTCCCGTGCT
GGACTCCGACGGCTCCTTCTTCCTCTACAGCAAGCTCACCGTGGACAAGAGCAGGTGGCAGCAGGGGAACGTCTTCTCATGCTCCGTGATGCATG
AGGCTCTGCACAACCACTACACGCAGAAGTCCCTCTCCCTGTCTCCGGGTAAATAG
>2130-1-0104-015_heavy_chain Redesigned COV2-2130 candidate 2130-1-0104-015 heavy chain
GAAGTGCAACTGGTGGAGAGCGGCGGGGGCCTTGTGAAGCCCGGCGGCTCCCTGCGCTTGTCATGCGCGGCCTCCGGTTTTACCTTCCGGGACGT
GTGGATGAGCTGGGTCAGACAGGCCCCTGGAAAAGGGCTGGAATGGGTCGGACGCATCAAGTCCAAGATCGACGGGGGAACGACAGACTACGCAG
CTCCAGTGAAGGGCCGGTTCACCATTAGCCGGGACGACTCGAAGAACACCCTGTATCTCCAAATGAACTCCCTCAAGACCGAAGATACCGCCGTG
TACTACTGCACTACCGCCGGTTCCTACTACTACGATACTGTGGGCCCGGGACTGCCCGAGGGAAAGTTCGATTACTGGGGACAGGGAACCCTGGT
CACTGTGTCGTCCGCCAGCACCAAGGGCCCATCGGTCTTCCCCCTGGCACCCTCCTCCAAGAGCACCTCTGGGGGCACAGCGGCCCTGGGCTGCC
TGGTCAAGGACTACTTCCCCGAACCGGTGACGGTGTCGTGGAACTCAGGCGCCCTGACCAGCGGCGTGCACACCTTCCCGGCTGTCCTACAGTCC
TCAGGACTCTACTCCCTCAGCAGCGTGGTGACCGTGCCCTCCAGCAGCTTGGGCACCCAGACCTACATCTGCAACGTGAATCACAAGCCCAGCAA
CACCAAGGTGGACAAGAAAGTTGAGCCCAAATCTTGTGACAAAACTCACACATGCCCACCGTGCCCAGCACCTGAACTCCTGGGGGGACCGTCAG
TCTTCCTCTTCCCCCCAAAACCCAAGGACACCCTCATGATCTCCCGGACCCCTGAGGTCACATGCGTGGTGGTGGACGTGAGCCACGAAGACCCT
GAGGTCAAGTTCAACTGGTACGTGGACGGCGTGGAGGTGCATAATGCCAAGACAAAGCCGCGGGAGGAGCAGTACAACAGCACGTACCGGGTGGT
CAGCGTCCTCACCGTCCTGCACCAGGACTGGCTGAATGGCAAGGAGTACAAGTGCAAGGTCAGCAACAAAGCCCTCCCAGCCCCCATCGAGAAAA
CCATCTCCAAAGCCAAAGGGCAGCCCCGAGAACCACAGGTGTACACCCTGCCCCCATCCCGGGATGAGCTGACCAAGAACCAGGTCAGCCTGACC
TGCCTGGTCAAAGGCTTCTATCCCAGCGACATCGCCGTGGAGTGGGAGAGCAATGGGCAGCCGGAGAACAACTACAAGACCACGCCTCCCGTGCT
GGACTCCGACGGCTCCTTCTTCCTCTACAGCAAGCTCACCGTGGACAAGAGCAGGTGGCAGCAGGGGAACGTCTTCTCATGCTCCGTGATGCATG
AGGCTCTGCACAACCACTACACGCAGAAGTCCCTCTCCCTGTCTCCGGGTAAATAG
>2130-1-0104-024_heavy_chain Redesigned COV2-2130 candidate 2130-1-0104-024 heavy chain
GAAGTGCAACTGGTGGAGAGCGGCGGGGGCCTTGTGAAGCCCGGCGGCTCCCTGCGCTTGTCATGCGCGGCCTCCGGTTTTACCTTCCGGGACGT
GTGGATGAGCTGGGTCAGACAGGCCCCTGGAAAAGGGCTGGAATGGGTCGGACGCATCAAGTCCAAGATCGACGGGGGAACGACAGACTACGCAG
```

```
CTCCAGTGAAGGGCCGGTTCACCATTAGCCGGGACGACTCGAAGAACACCCTGTATCTCCAAATGAACTCCCTCAAGACCGAAGATACCGCCGTG
TACTACTGCACTACCGCCGGTTCCTACTACTACGATACTGTGGGCCCGGGACTGCCCGAGGGAAAGTTCGATTACTGGGGACAGGGAACCCTGGT
CACTGTGTCGTCCGCCAGCACCAAGGGCCCATCGGTCTTCCCCCTGGCACCCTCCTCCAAGAGCACCTCTGGGGGCACAGCGGCCCTGGGCTGCC
TGGTCAAGGACTACTTCCCCGAACCGGTGACGGTGTCGTGGAACTCAGGCGCCCTGACCAGCGGCGTGCACACCTTCCCGGCTGTCCTACAGTCC
TCAGGACTCTACTCCCTCAGCAGCGTGGTGACCGTGCCCTCCAGCAGCTTGGGCACCCAGACCTACATCTGCAACGTGAATCACAAGCCCAGCAA
CACCAAGGTGGACAAGAAAGTTGAGCCCAAATCTTGTGACAAAACTCACACATGCCCACCGTGCCCAGCACCTGAACTCCTGGGGGGACCGTCAG
TCTTCCTCTTCCCCCCAAAACCCAAGGACACCCTCATGATCTCCCGGACCCCTGAGGTCACATGCGTGGTGGTGGACGTGAGCCACGAAGACCCT
GAGGTCAAGTTCAACTGGTACGTGGACGGCGTGGAGGTGCATAATGCCAAGACAAAGCCGCGGGAGGAGCAGTACAACAGCACGTACCGGGTGGT
CAGCGTCCTCACCGTCCTGCACCAGGACTGGCTGAATGGCAAGGAGTACAAGTGCAAGGTCAGCAACAAAGCCCTCCCAGCCCCCATCGAGAAAA
CCATCTCCAAAGCCAAAGGGCAGCCCCGAGAACCACAGGTGTACACCCTGCCCCCATCCCGGGATGAGCTGACCAAGAACCAGGTCAGCCTGACC
TGCCTGGTCAAAGGCTTCTATCCCAGCGACATCGCCGTGGAGTGGGAGAGCAATGGGCAGCCGGAGAACAACTACAAGACCACGCCTCCCGTGCT
GGACTCCGACGGCTCCTTCTTCCTCTACAGCAAGCTCACCGTGGACAAGAGCAGGTGGCAGCAGGGGAACGTCTTCTCATGCTCCGTGATGCATG
AGGCTCTGCACAACCACTACACGCAGAAGTCCCTCTCCCTGTCTCCGGGTAAATAG
>2130-1-0111-002_heavy_chain Redesigned COV2-2130 candidate 2130-1-0111-002 heavy chain
GAAGTGCAGCTGGTCGAATCCGGTGGCGGCCTCGTGAAGCCTGGGGGAAGCCTGCGCTTGTCGTGTGCCGCGAGCGGATTCACTTTTCGGGACGT
GTGGATGTCCTGGGTCCGCCAGGCACCGGGAAAGGGACTGGAATGGGTCGGCAGGATTAAGTCGAAAGACGATGGCGGAACGACCGACTACGCCG
CCCCCGTGAAGGGCAGATTCACCATCTCCCGGGACGATTCCAAGAACACCCTGTACCTCCAAATGAACTCCCTTAAGACCGAGGACACTGCTGTG
TACTATTGCACCACCGCCGGGTCATACTACTACGATACTGTGGGCCCGGGTCTGCCAGAGGGAAAGTTCGACTACTGGGGACAGGGGACCCTGGT
CACTGTGTCCAGCGCCAGCACCAAGGGCCCATCGGTCTTCCCCCTGGCACCCTCCTCCAAGAGCACCTCTGGGGGCACAGCGGCCCTGGGCTGCC
TGGTCAAGGACTACTTCCCCGAACCGGTGACGGTGTCGTGGAACTCAGGCGCCCTGACCAGCGGCGTGCACACCTTCCCGGCTGTCCTACAGTCC
TCAGGACTCTACTCCCTCAGCAGCGTGGTGACCGTGCCCTCCAGCAGCTTGGGCACCCAGACCTACATCTGCAACGTGAATCACAAGCCCAGCAA
CACCAAGGTGGACAAGAAAGTTGAGCCCAAATCTTGTGACAAAACTCACACATGCCCACCGTGCCCAGCACCTGAACTCCTGGGGGGACCGTCAG
TCTTCCTCTTCCCCCCAAAACCCAAGGACACCCTCATGATCTCCCGGACCCCTGAGGTCACATGCGTGGTGGTGGACGTGAGCCACGAAGACCCT
GAGGTCAAGTTCAACTGGTACGTGGACGGCGTGGAGGTGCATAATGCCAAGACAAAGCCGCGGGAGGAGCAGTACAACAGCACGTACCGGGTGGT
CAGCGTCCTCACCGTCCTGCACCAGGACTGGCTGAATGGCAAGGAGTACAAGTGCAAGGTCAGCAACAAAGCCCTCCCAGCCCCCATCGAGAAAA
CCATCTCCAAAGCCAAAGGGCAGCCCCGAGAACCACAGGTGTACACCCTGCCCCCATCCCGGGATGAGCTGACCAAGAACCAGGTCAGCCTGACC
TGCCTGGTCAAAGGCTTCTATCCCAGCGACATCGCCGTGGAGTGGGAGAGCAATGGGCAGCCGGAGAACAACTACAAGACCACGCCTCCCGTGCT
GGACTCCGACGGCTCCTTCTTCCTCTACAGCAAGCTCACCGTGGACAAGAGCAGGTGGCAGCAGGGGAACGTCTTCTCATGCTCCGTGATGCATG
AGGCTCTGCACAACCACTACACGCAGAAGTCCCTCTCCCTGTCTCCGGGTAAATAG
>2130-1-0114-111_heavy_chain Redesigned COV2-2130 candidate 2130-1-0114-111 heavy chain
GAAGTGCAGCTCGTGGAGTCCGGTGGCGGACTGGTCAAGCCTGGCGGATCATTGCGGCTGTCCTGTGCGGCATCCGGATTCACTTTCCGGGACGT
GTGGATGAGCTGGGTCCGCCAGGCCCCGGGAAAGGGACTGGAATGGGTCGGCAGAATCAAGTCCAAGATTGACGGCGGGACTACCGATTACGCCG
CCCCAGTGAAGGGTCGCTTCACTATCTCGAGGGACGACAGCAAAAACACGCTGTACCTCCAAATGAACTCCCTCAAGACCGAGGACACCGCCGTG
TACTACTGCACCACCGCTGGAAGCTACTACTACGATACTGTGGGGCCGGAACTGCCCGAGGGAAAGTTTGATTATTGGGGCCAGGGGACCCTTGT
GACCGTGTCCTCGGCCAGCACCAAGGGCCCATCGGTCTTCCCCCTGGCACCCTCCTCCAAGAGCACCTCTGGGGGCACAGCGGCCCTGGGCTGCC
TGGTCAAGGACTACTTCCCCGAACCGGTGACGGTGTCGTGGAACTCAGGCGCCCTGACCAGCGGCGTGCACACCTTCCCGGCTGTCCTACAGTCC
TCAGGACTCTACTCCCTCAGCAGCGTGGTGACCGTGCCCTCCAGCAGCTTGGGCACCCAGACCTACATCTGCAACGTGAATCACAAGCCCAGCAA
CACCAAGGTGGACAAGAAAGTTGAGCCCAAATCTTGTGACAAAACTCACACATGCCCACCGTGCCCAGCACCTGAACTCCTGGGGGGACCGTCAG
TCTTCCTCTTCCCCCCAAAACCCAAGGACACCCTCATGATCTCCCGGACCCCTGAGGTCACATGCGTGGTGGTGGACGTGAGCCACGAAGACCCT
GAGGTCAAGTTCAACTGGTACGTGGACGGCGTGGAGGTGCATAATGCCAAGACAAAGCCGCGGGAGGAGCAGTACAACAGCACGTACCGGGTGGT
CAGCGTCCTCACCGTCCTGCACCAGGACTGGCTGAATGGCAAGGAGTACAAGTGCAAGGTCAGCAACAAAGCCCTCCCAGCCCCCATCGAGAAAA
CCATCTCCAAAGCCAAAGGGCAGCCCCGAGAACCACAGGTGTACACCCTGCCCCCATCCCGGGATGAGCTGACCAAGAACCAGGTCAGCCTGACC
TGCCTGGTCAAAGGCTTCTATCCCAGCGACATCGCCGTGGAGTGGGAGAGCAATGGGCAGCCGGAGAACAACTACAAGACCACGCCTCCCGTGCT
GGACTCCGACGGCTCCTTCTTCCTCTACAGCAAGCTCACCGTGGACAAGAGCAGGTGGCAGCAGGGGAACGTCTTCTCATGCTCCGTGATGCATG
AGGCTCTGCACAACCACTACACGCAGAAGTCCCTCTCCCTGTCTCCGGGTAAATAG
>2130-1-0114-112_heavy_chain Redesigned COV2-2130 candidate 2130-1-0114-112 heavy chain
GAAGTGCAGCTCGTGGAGTCCGGTGGCGGACTGGTCAAGCCTGGCGGATCATTGCGGCTGTCCTGTGCGGCATCCGGATTCACTTTCCGGGACGT
GTGGATGAGCTGGGTCCGCCAGGCCCCGGGAAAGGGACTGGAATGGGTCGGCAGAATCAAGTCCAAGATTGACGGCGGGACTACCGATTACGCCG
CCCCAGTGAAGGGTCGCTTCACTATCTCGAGGGACGACAGCAAAAACACGCTGTACCTCCAAATGAACTCCCTCAAGACCGAGGACACCGCCGTG
TACTACTGCACCACCGCTGGAAGCTACTACTACGATACTGTGGGGCCGGAACTGCCCGAGGGAAAGTTTGATTATTGGGGCCAGGGGACCCTTGT
GACCGTGTCCTCGGCCAGCACCAAGGGCCCATCGGTCTTCCCCCTGGCACCCTCCTCCAAGAGCACCTCTGGGGGCACAGCGGCCCTGGGCTGCC
TGGTCAAGGACTACTTCCCCGAACCGGTGACGGTGTCGTGGAACTCAGGCGCCCTGACCAGCGGCGTGCACACCTTCCCGGCTGTCCTACAGTCC
TCAGGACTCTACTCCCTCAGCAGCGTGGTGACCGTGCCCTCCAGCAGCTTGGGCACCCAGACCTACATCTGCAACGTGAATCACAAGCCCAGCAA
CACCAAGGTGGACAAGAAAGTTGAGCCCAAATCTTGTGACAAAACTCACACATGCCCACCGTGCCCAGCACCTGAACTCCTGGGGGGACCGTCAG
TCTTCCTCTTCCCCCCAAAACCCAAGGACACCCTCATGATCTCCCGGACCCCTGAGGTCACATGCGTGGTGGTGGACGTGAGCCACGAAGACCCT
GAGGTCAAGTTCAACTGGTACGTGGACGGCGTGGAGGTGCATAATGCCAAGACAAAGCCGCGGGAGGAGCAGTACAACAGCACGTACCGGGTGGT
CAGCGTCCTCACCGTCCTGCACCAGGACTGGCTGAATGGCAAGGAGTACAAGTGCAAGGTCAGCAACAAAGCCCTCCCAGCCCCCATCGAGAAAA
CCATCTCCAAAGCCAAAGGGCAGCCCCGAGAACCACAGGTGTACACCCTGCCCCCATCCCGGGATGAGCTGACCAAGAACCAGGTCAGCCTGACC
TGCCTGGTCAAAGGCTTCTATCCCAGCGACATCGCCGTGGAGTGGGAGAGCAATGGGCAGCCGGAGAACAACTACAAGACCACGCCTCCCGTGCT
GGACTCCGACGGCTCCTTCTTCCTCTACAGCAAGCTCACCGTGGACAAGAGCAGGTGGCAGCAGGGGAACGTCTTCTCATGCTCCGTGATGCATG
AGGCTCTGCACAACCACTACACGCAGAAGTCCCTCTCCCTGTCTCCGGGTAAATAG
```

Supplementary Table 5 | Selected sequence records as constructed: IgG light-chain DNA sequences

```
>2130-1-1231-017_light_chain Redesigned COV2-2130 candidate 2130-1-1231-017 light chain
GATATTGTGATGACTCAGTCACCCGACTCGCTCGCCGTGTCACTGGGGGAGAGAGCCACCATCAACTGCAAAAGCTCCCAGAGCGTGCTGTATTG
GGCCAACAACAAGAATTACCTCGCGTGGTACCAGCAGAAGCCTGGACAGCCGCCAAAGCTGCTTATGTACTGGGCTTCGACCCGCGAATCCGGAG
TGCCGGACCGGTTTAGCGGCTCCGGGTCCGGAGCCGAATTCACCCTGACCATTTCCTCGTTGCAAGCCGAGGACGTGGCAATCTACTACTGTCAA
CAGTACTACTCCACTCTGACTTTCGGTGGCGGCACCAAGGTCGAAATCAAGCGAACTGTGGCTGCACCATCTGTCTTCATCTTCCCGCCATCTGA
TGAGCAGTTGAAATCTGGAACTGCCTCTGTTGTGTGCCTGCTGAATAACTTCTATCCCAGAGAGGCCAAAGTACAGTGGAAGGTGGATAACGCCC
TCCAATCGGGTAACTCCCAGGAGAGTGTCACAGAGCAGGACAGCAAGGACAGCACCTACAGCCTCAGCAGCACCCTGACGCTGAGCAAAGCAGAC
TACGAGAAACACAAAGTCTACGCCTGCGAAGTCACCCATCAGGGCCTGAGCTCGCCCGTCACAAAGAGCTTCAACAGGGGAGAGTGTTAA

>2130-1-1231-174_light_chain Redesigned COV2-2130 candidate 2130-1-1231-174 light chain
GACATTGTGATGACTCAGTCCCCCGACTCACTGGCCGTCAGCCTTGGAGAGCGCGCTACCATCAACTGCAAAAGCTCCCAGAGCGTGCTGTACTC
CTCCAACAACAAGAATTACCTCGCCTGGTACCAACAGAAGCCTGGCCAGCCACCGAAGCTGCTGATGTACTGGGCATCCACTAGAGAGTCGGGAG
TGCCGGACCGGTTTTCCGGTTCCGGATCGGGGGCCGAGTTCACTCTGACCATTTCGAGCCTCCAAGCGGAAGATGTGGCCATCTACTACTGTCAG
CAGTATTACTCAACCTTGACCTTCGGGGGCGGCACCAAGGTCGAAATCAAGCGAACTGTGGCTGCACCATCTGTCTTCATCTTCCCGCCATCTGA
TGAGCAGTTGAAATCTGGAACTGCCTCTGTTGTGTGCCTGCTGAATAACTTCTATCCCAGAGAGGCCAAAGTACAGTGGAAGGTGGATAACGCCC
TCCAATCGGGTAACTCCCAGGAGAGTGTCACAGAGCAGGACAGCAAGGACAGCACCTACAGCCTCAGCAGCACCCTGACGCTGAGCAAAGCAGAC
TACGAGAAACACAAAGTCTACGCCTGCGAAGTCACCCATCAGGGCCTGAGCTCGCCCGTCACAAAGAGCTTCAACAGGGGAGAGTGTTAA

>2130-1-1231-200_light_chain Redesigned COV2-2130 candidate 2130-1-1231-200 light chain
GACATCGTGATGACCCAGAGCCCGGACTCACTGGCCGTCAGCCTCGGAGAGCGCGCGACCATTAATTGCAAAAGCTCCCAGTCCGTGCTGTACTC
ATGGAACAACAAGAACTACCTTGCCTGGTATCAGCAGAAGCCTGGGCAACCGCCAAAGCTGTTGATGTACTGGGCATCCACCAGAGAATCCGGCG
TGCCCGACCGGTTCTCGGGTTCCGGATCCGGCGCTGAGTTTACCCTGACTATCTCGTCGCTGCAAGCCGAAGATGTGGCCATCTACTACTGTCAG
CAGTACTACTCCACTCTCACTTTCGGGGGCGGAACCAAGGTCGAGATTAAGCGAACTGTGGCTGCACCATCTGTCTTCATCTTCCCGCCATCTGA
TGAGCAGTTGAAATCTGGAACTGCCTCTGTTGTGTGCCTGCTGAATAACTTCTATCCCAGAGAGGCCAAAGTACAGTGGAAGGTGGATAACGCCC
TCCAATCGGGTAACTCCCAGGAGAGTGTCACAGAGCAGGACAGCAAGGACAGCACCTACAGCCTCAGCAGCACCCTGACGCTGAGCAAAGCAGAC
TACGAGAAACACAAAGTCTACGCCTGCGAAGTCACCCATCAGGGCCTGAGCTCGCCCGTCACAAAGAGCTTCAACAGGGGAGAGTGTTAA

>2130-1-0104-015_light_chain Redesigned COV2-2130 candidate 2130-1-0104-015 light chain
GATATCGTGATGACCCAGTCGCCGGACTCGCTTGCCGTGTCATTGGGGGAGAGAGCGACTATCAACTGCAAGAGCAGCCAGTCCGTGCTGTACCA
GTACAACAACAAGAATTATCTGGCCTGGTACCAGCAGAAGCCCGGACAGCCTCCGAAGCTGCTGATGTACTGGGCCAGCCACCGGGAATCCGGCG
TGCCAGACCGCTTTTCCGGATCCGGCTCGGGAGCCGAATTCACCCTCACCATTTCATCCCTGCAAGCTGAGGACGTGGCAATCTACTACTGCCAA
CAGTACTACTCCACCCTCACTTTCGGGGGTGGCACCAAAGTCGAGATTAAGCGAACTGTGGCTGCACCATCTGTCTTCATCTTCCCGCCATCTGA
TGAGCAGTTGAAATCTGGAACTGCCTCTGTTGTGTGCCTGCTGAATAACTTCTATCCCAGAGAGGCCAAAGTACAGTGGAAGGTGGATAACGCCC
TCCAATCGGGTAACTCCCAGGAGAGTGTCACAGAGCAGGACAGCAAGGACAGCACCTACAGCCTCAGCAGCACCCTGACGCTGAGCAAAGCAGAC
TACGAGAAACACAAAGTCTACGCCTGCGAAGTCACCCATCAGGGCCTGAGCTCGCCCGTCACAAAGAGCTTCAACAGGGGAGAGTGTTAA

>2130-1-0104-024_light_chain Redesigned COV2-2130 candidate 2130-1-0104-024 light chain
GATATTGTGATGACCCAGTCCCCTGACTCGCTGGCCGTGTCGCTTGGGGAGAGAGCAACCATCAATTGCAAGAGCAGCCAGTCCGTGCTGTACTG
GTCCAACAACAAGAACTACCTGGCCTGGTACCAACAGAAACCCGGACAGCCGCCGAAGCTCCTGATGTACTGGGCCTCAGAGCGGGAATCCGGAG
TGCCAGACCGCTTTTCCGGATCCGGCAGCGGTGCCGAATTCACCCTGACTATTTCGTCACTCCAAGCTGAGGACGTGGCGATCTACTACTGTCAG
CAGTACTATTCCACTTTGACCTTCGGCGGCGGGACCAAGGTCGAAATCAAGCGAACTGTGGCTGCACCATCTGTCTTCATCTTCCCGCCATCTGA
TGAGCAGTTGAAATCTGGAACTGCCTCTGTTGTGTGCCTGCTGAATAACTTCTATCCCAGAGAGGCCAAAGTACAGTGGAAGGTGGATAACGCCC
TCCAATCGGGTAACTCCCAGGAGAGTGTCACAGAGCAGGACAGCAAGGACAGCACCTACAGCCTCAGCAGCACCCTGACGCTGAGCAAAGCAGAC
TACGAGAAACACAAAGTCTACGCCTGCGAAGTCACCCATCAGGGCCTGAGCTCGCCCGTCACAAAGAGCTTCAACAGGGGAGAGTGTTAA

>2130-1-0111-002_light_chain Redesigned COV2-2130 candidate 2130-1-0111-002 light chain
GATATTGTGATGACCCAGAGCCCGGACAGCCTGGCCGTGTCCCTCGGCGAACGCGCTACCATTAACTGCAAATCATCACAGTCCGTGCTGTATTC
GTTCAACAACAAGAATTACCTGGCCTGGTACCAGCAGAAGCCCGGGCAGCCTCCGAAGCTCCTGATGTACTGGGCCTCCGAACGGGAGAGCGGAG
TGCCAGACAGATTCTCGGGCTCCGGGTCCGGAGCGGAATTCACCCTTACTATCTCCTCGTTGCAAGCCGAGGACGTGGCAATCTACTACTGCCAA
CAGTACTACTCCACCCTGACCTTTGGTGGCGGAACTAAGGTCGAGATCAAGCGAACTGTGGCTGCACCATCTGTCTTCATCTTCCCGCCATCTGA
TGAGCAGTTGAAATCTGGAACTGCCTCTGTTGTGTGCCTGCTGAATAACTTCTATCCCAGAGAGGCCAAAGTACAGTGGAAGGTGGATAACGCCC
TCCAATCGGGTAACTCCCAGGAGAGTGTCACAGAGCAGGACAGCAAGGACAGCACCTACAGCCTCAGCAGCACCCTGACGCTGAGCAAAGCAGAC
TACGAGAAACACAAAGTCTACGCCTGCGAAGTCACCCATCAGGGCCTGAGCTCGCCCGTCACAAAGAGCTTCAACAGGGGAGAGTGTTAA

>2130-1-0114-111_light_chain Redesigned COV2-2130 candidate 2130-1-0114-111 light chain
GACATTGTGATGACTCAGTCACCTGATTCGTTGGCCGTGTCCCTGGGAGAACGGGCAACCATTAACTGCAAGAGCAGCCAGTCAGTCCTGTACTA
CGTGAACAACTACAATTATCTGGCCTGGTACCAGCAGAAGCCCGGGCAGCCACCGAAGCTTCTCATGTACTGGGCGTCCACCCGCGAATCCGGAG
TGCCGGACAGATTTTCCGGGTCGGGAAGCGGCGCTGAATTCACCCTGACTATCTCCTCGCTGCAAGCCGAGGACGTGGCCATCTACTACTGCCAA
CAGTACTACTCCACTCTCACCTTCGGTGGCGGCACCAAAGTCGAGATCAAGCGAACTGTGGCTGCACCATCTGTCTTCATCTTCCCGCCATCTGA
TGAGCAGTTGAAATCTGGAACTGCCTCTGTTGTGTGCCTGCTGAATAACTTCTATCCCAGAGAGGCCAAAGTACAGTGGAAGGTGGATAACGCCC
TCCAATCGGGTAACTCCCAGGAGAGTGTCACAGAGCAGGACAGCAAGGACAGCACCTACAGCCTCAGCAGCACCCTGACGCTGAGCAAAGCAGAC
TACGAGAAACACAAAGTCTACGCCTGCGAAGTCACCCATCAGGGCCTGAGCTCGCCCGTCACAAAGAGCTTCAACAGGGGAGAGTGTTAA
```

```
>2130-1-0114-112_light_chain Redesigned COV2-2130 candidate 2130-1-0114-112 light chain
GACATCGTGATGACTCAATCGCCCGACTCACTCGCCGTGTCCTTGGGGGAACGGGCCACCATCAATTGCAAGAGCAGCCAGTCCGTGCTGTACGC
CGCAAACAACAAGAACTATCTCGCTTGGTACCAGCAGAAGCCTGGACAGCCGCCAAAACTTCTGATGTACTGGGCGTCGGAGCGCGAGTCCGGAG
TGCCGGACAGATTTTCCGGAAGCGGCTCCGGCGCCGAATTCACCCTGACCATTTCCTCACTGCAAGCCGAAGATGTGGCGATCTACTACTGCCAG
CAGTACTACTCGACCCTGACTTTCGGTGGCGGGACCAAGGTCGAGATTAAGCGAACTGTGGCTGCACCATCTGTCTTCATCTTCCCGCCATCTGA
TGAGCAGTTGAAATCTGGAACTGCCTCTGTTGTGTGCCTGCTGAATAACTTCTATCCCAGAGAGGCCAAAGTACAGTGGAAGGTGGATAACGCCC
TCCAATCGGGTAACTCCCAGGAGAGTGTCACAGAGCAGGACAGCAAGGACAGCACCTACAGCCTCAGCAGCACCCTGACGCTGAGCAAAGCAGAC
TACGAGAAACACAAAGTCTACGCCTGCGAAGTCACCCATCAGGGCCTGAGCTCGCCCGTCACAAAGAGCTTCAACAGGGGAGAGTGTTAA
```

Supplementary Table 6 | Selected sequence records as constructed: IgG heavy-chain amino acid sequences

>2130-1-1231-017_heavy_chain
EVQLVESGGGLVKPGGSLRLSCAASGFTFRDVWMSWVRQAPGKGLEWVGRIKSKEDGGTTDYAAPVKGRFTISRDDSKNTLYLQMNSLKTEDTAV
YYCTTAGSYYFDTVGPGLPEGKFDYWGQGTLVTVSSASTKGPSVFPLAPSSKSTSGGTAALGCLVKDYFPEPVTVSWNSGALTSGVHTFPAVLQS
SGLYSLSSVVTVPSSSLGTQTYICNVNHKPSNTKVDKKVEPKSCDKTHTCPPCPAPELLGGPSVFLFPPKPKDTLMISRTPEVTCVVVDVSHEDP
EVKFNWYVDGVEVHNAKTKPREEQYNSTYRVVSVLTVLHQDWLNGKEYKCKVSNKALPAPIEKTISKAKGQPREPQVYTLPPSRDELTKNQVSLT
CLVKGFYPSDIAVEWESNGQPENNYKTTPPVLDSDGSFFLYSKLTVDKSRWQQGNVFSCSVMHEALHNHYTQKSLSLSPGK*

>2130-1-1231-174_heavy_chain
EVQLVESGGGLVKPGGSLRLSCAASGFTFRDVWMSWVRQAPGKGLEWVGRIKSKIDGGTTDYAAPVKGRFTISRDDSKNTLYLQMNSLKTEDTAV
YYCTTAGSYYYDTRGPGLPEGKFDYWGQGTLVTVSSASTKGPSVFPLAPSSKSTSGGTAALGCLVKDYFPEPVTVSWNSGALTSGVHTFPAVLQS
SGLYSLSSVVTVPSSSLGTQTYICNVNHKPSNTKVDKKVEPKSCDKTHTCPPCPAPELLGGPSVFLFPPKPKDTLMISRTPEVTCVVVDVSHEDP
EVKFNWYVDGVEVHNAKTKPREEQYNSTYRVVSVLTVLHQDWLNGKEYKCKVSNKALPAPIEKTISKAKGQPREPQVYTLPPSRDELTKNQVSLT
CLVKGFYPSDIAVEWESNGQPENNYKTTPPVLDSDGSFFLYSKLTVDKSRWQQGNVFSCSVMHEALHNHYTQKSLSLSPGK*

>2130-1-1231-200_heavy_chain
EVQLVESGGGLVKPGGSLRLSCAASGFTFRDVWMSWVRQAPGKGLEWVGRIKSKIDGGTTDYAAPVKGRFTISRDDSKNTLYLQMNSLKTEDTAV
YYCTTAGSYYYDTVGPGLPEGKFDYWGQGTLVTVSSASTKGPSVFPLAPSSKSTSGGTAALGCLVKDYFPEPVTVSWNSGALTSGVHTFPAVLQS
SGLYSLSSVVTVPSSSLGTQTYICNVNHKPSNTKVDKKVEPKSCDKTHTCPPCPAPELLGGPSVFLFPPKPKDTLMISRTPEVTCVVVDVSHEDP
EVKFNWYVDGVEVHNAKTKPREEQYNSTYRVVSVLTVLHQDWLNGKEYKCKVSNKALPAPIEKTISKAKGQPREPQVYTLPPSRDELTKNQVSLT
CLVKGFYPSDIAVEWESNGQPENNYKTTPPVLDSDGSFFLYSKLTVDKSRWQQGNVFSCSVMHEALHNHYTQKSLSLSPGK*

>2130-1-0104-015_heavy_chain
EVQLVESGGGLVKPGGSLRLSCAASGFTFRDVWMSWVRQAPGKGLEWVGRIKSKIDGGTTDYAAPVKGRFTISRDDSKNTLYLQMNSLKTEDTAV
YYCTTAGSYYYDTVGPGLPEGKFDYWGQGTLVTVSSASTKGPSVFPLAPSSKSTSGGTAALGCLVKDYFPEPVTVSWNSGALTSGVHTFPAVLQS
SGLYSLSSVVTVPSSSLGTQTYICNVNHKPSNTKVDKKVEPKSCDKTHTCPPCPAPELLGGPSVFLFPPKPKDTLMISRTPEVTCVVVDVSHEDP
EVKFNWYVDGVEVHNAKTKPREEQYNSTYRVVSVLTVLHQDWLNGKEYKCKVSNKALPAPIEKTISKAKGQPREPQVYTLPPSRDELTKNQVSLT
CLVKGFYPSDIAVEWESNGQPENNYKTTPPVLDSDGSFFLYSKLTVDKSRWQQGNVFSCSVMHEALHNHYTQKSLSLSPGK*

>2130-1-0104-024_heavy_chain
EVQLVESGGGLVKPGGSLRLSCAASGFTFRDVWMSWVRQAPGKGLEWVGRIKSKIDGGTTDYAAPVKGRFTISRDDSKNTLYLQMNSLKTEDTAV
YYCTTAGSYYYDTVGPGLPEGKFDYWGQGTLVTVSSASTKGPSVFPLAPSSKSTSGGTAALGCLVKDYFPEPVTVSWNSGALTSGVHTFPAVLQS
SGLYSLSSVVTVPSSSLGTQTYICNVNHKPSNTKVDKKVEPKSCDKTHTCPPCPAPELLGGPSVFLFPPKPKDTLMISRTPEVTCVVVDVSHEDP
EVKFNWYVDGVEVHNAKTKPREEQYNSTYRVVSVLTVLHQDWLNGKEYKCKVSNKALPAPIEKTISKAKGQPREPQVYTLPPSRDELTKNQVSLT
CLVKGFYPSDIAVEWESNGQPENNYKTTPPVLDSDGSFFLYSKLTVDKSRWQQGNVFSCSVMHEALHNHYTQKSLSLSPGK*

>2130-1-0111-002_heavy_chain
EVQLVESGGGLVKPGGSLRLSCAASGFTFRDVWMSWVRQAPGKGLEWVGRIKSKDDGGTTDYAAPVKGRFTISRDDSKNTLYLQMNSLKTEDTAV
YYCTTAGSYYYDTVGPGLPEGKFDYWGQGTLVTVSSASTKGPSVFPLAPSSKSTSGGTAALGCLVKDYFPEPVTVSWNSGALTSGVHTFPAVLQS
SGLYSLSSVVTVPSSSLGTQTYICNVNHKPSNTKVDKKVEPKSCDKTHTCPPCPAPELLGGPSVFLFPPKPKDTLMISRTPEVTCVVVDVSHEDP
EVKFNWYVDGVEVHNAKTKPREEQYNSTYRVVSVLTVLHQDWLNGKEYKCKVSNKALPAPIEKTISKAKGQPREPQVYTLPPSRDELTKNQVSLT
CLVKGFYPSDIAVEWESNGQPENNYKTTPPVLDSDGSFFLYSKLTVDKSRWQQGNVFSCSVMHEALHNHYTQKSLSLSPGK*

>2130-1-0114-111_heavy_chain
EVQLVESGGGLVKPGGSLRLSCAASGFTFRDVWMSWVRQAPGKGLEWVGRIKSKIDGGTTDYAAPVKGRFTISRDDSKNTLYLQMNSLKTEDTAV
YYCTTAGSYYYDTVGPELPEGKFDYWGQGTLVTVSSASTKGPSVFPLAPSSKSTSGGTAALGCLVKDYFPEPVTVSWNSGALTSGVHTFPAVLQS
SGLYSLSSVVTVPSSSLGTQTYICNVNHKPSNTKVDKKVEPKSCDKTHTCPPCPAPELLGGPSVFLFPPKPKDTLMISRTPEVTCVVVDVSHEDP
EVKFNWYVDGVEVHNAKTKPREEQYNSTYRVVSVLTVLHQDWLNGKEYKCKVSNKALPAPIEKTISKAKGQPREPQVYTLPPSRDELTKNQVSLT
CLVKGFYPSDIAVEWESNGQPENNYKTTPPVLDSDGSFFLYSKLTVDKSRWQQGNVFSCSVMHEALHNHYTQKSLSLSPGK*

>2130-1-0114-112_heavy_chain
EVQLVESGGGLVKPGGSLRLSCAASGFTFRDVWMSWVRQAPGKGLEWVGRIKSKIDGGTTDYAAPVKGRFTISRDDSKNTLYLQMNSLKTEDTAV
YYCTTAGSYYYDTVGPELPEGKFDYWGQGTLVTVSSASTKGPSVFPLAPSSKSTSGGTAALGCLVKDYFPEPVTVSWNSGALTSGVHTFPAVLQS
SGLYSLSSVVTVPSSSLGTQTYICNVNHKPSNTKVDKKVEPKSCDKTHTCPPCPAPELLGGPSVFLFPPKPKDTLMISRTPEVTCVVVDVSHEDP
EVKFNWYVDGVEVHNAKTKPREEQYNSTYRVVSVLTVLHQDWLNGKEYKCKVSNKALPAPIEKTISKAKGQPREPQVYTLPPSRDELTKNQVSLT
CLVKGFYPSDIAVEWESNGQPENNYKTTPPVLDSDGSFFLYSKLTVDKSRWQQGNVFSCSVMHEALHNHYTQKSLSLSPGK*

Supplementary Table 7 | Selected sequence records as constructed: IgG light-chain amino acid sequences

```
>2130-1-1231-017_light_chain
DIVMTQSPDSLAVSLGERATINCKSSQSVLYWANNKNYLAWYQQKPGQPPKLLMYWASTRESGVPDRFSGSGSGAEFTLTISSLQAEDVAIYYCQ
QYYSTLTFGGGTKVEIKRTVAAPSVFIFPPSDEQLKSGTASVVCLLNNFYPREAKVQWKVDNALQSGNSQESVTEQDSKDSTYSLSSTLTLSKAD
YEKHKVYACEVTHQGLSSPVTKSFNRGEC*

>2130-1-1231-174_light_chain
DIVMTQSPDSLAVSLGERATINCKSSQSVLYSSNNKNYLAWYQQKPGQPPKLLMYWASTRESGVPDRFSGSGSGAEFTLTISSLQAEDVAIYYCQ
QYYSTLTFGGGTKVEIKRTVAAPSVFIFPPSDEQLKSGTASVVCLLNNFYPREAKVQWKVDNALQSGNSQESVTEQDSKDSTYSLSSTLTLSKAD
YEKHKVYACEVTHQGLSSPVTKSFNRGEC*

>2130-1-1231-200_light_chain
DIVMTQSPDSLAVSLGERATINCKSSQSVLYSWNNKNYLAWYQQKPGQPPKLLMYWASTRESGVPDRFSGSGSGAEFTLTISSLQAEDVAIYYCQ
QYYSTLTFGGGTKVEIKRTVAAPSVFIFPPSDEQLKSGTASVVCLLNNFYPREAKVQWKVDNALQSGNSQESVTEQDSKDSTYSLSSTLTLSKAD
YEKHKVYACEVTHQGLSSPVTKSFNRGEC*

>2130-1-0104-015_light_chain
DIVMTQSPDSLAVSLGERATINCKSSQSVLYQYNNKNYLAWYQQKPGQPPKLLMYWASHRESGVPDRFSGSGSGAEFTLTISSLQAEDVAIYYCQ
QYYSTLTFGGGTKVEIKRTVAAPSVFIFPPSDEQLKSGTASVVCLLNNFYPREAKVQWKVDNALQSGNSQESVTEQDSKDSTYSLSSTLTLSKAD
YEKHKVYACEVTHQGLSSPVTKSFNRGEC*

>2130-1-0104-024_light_chain
DIVMTQSPDSLAVSLGERATINCKSSQSVLYWSNNKNYLAWYQQKPGQPPKLLMYWASERESGVPDRFSGSGSGAEFTLTISSLQAEDVAIYYCQ
QYYSTLTFGGGTKVEIKRTVAAPSVFIFPPSDEQLKSGTASVVCLLNNFYPREAKVQWKVDNALQSGNSQESVTEQDSKDSTYSLSSTLTLSKAD
YEKHKVYACEVTHQGLSSPVTKSFNRGEC*

>2130-1-0111-002_light_chain
DIVMTQSPDSLAVSLGERATINCKSSQSVLYSFNNKNYLAWYQQKPGQPPKLLMYWASERESGVPDRFSGSGSGAEFTLTISSLQAEDVAIYYCQ
QYYSTLTFGGGTKVEIKRTVAAPSVFIFPPSDEQLKSGTASVVCLLNNFYPREAKVQWKVDNALQSGNSQESVTEQDSKDSTYSLSSTLTLSKAD
YEKHKVYACEVTHQGLSSPVTKSFNRGEC*

>2130-1-0114-111_light_chain
DIVMTQSPDSLAVSLGERATINCKSSQSVLYYVNNYNYLAWYQQKPGQPPKLLMYWASTRESGVPDRFSGSGSGAEFTLTISSLQAEDVAIYYCQ
QYYSTLTFGGGTKVEIKRTVAAPSVFIFPPSDEQLKSGTASVVCLLNNFYPREAKVQWKVDNALQSGNSQESVTEQDSKDSTYSLSSTLTLSKAD
YEKHKVYACEVTHQGLSSPVTKSFNRGEC*

>2130-1-0114-112_light_chain
DIVMTQSPDSLAVSLGERATINCKSSQSVLYAANNKNYLAWYQQKPGQPPKLLMYWASERESGVPDRFSGSGSGAEFTLTISSLQAEDVAIYYCQ
QYYSTLTFGGGTKVEIKRTVAAPSVFIFPPSDEQLKSGTASVVCLLNNFYPREAKVQWKVDNALQSGNSQESVTEQDSKDSTYSLSSTLTLSKAD
YEKHKVYACEVTHQGLSSPVTKSFNRGEC*
```

Supplementary Table 8 | Cryo-EM data collection, refinement, and validation statistics

	(EMDB-28198)	(EMDB-28199)<br>(PDB 8EKD)
**Data collection and processing**		
Magnification	130,000	130,000
Voltage (kV)	300	300
Electron exposure (e–/Å^2)	52.2	52.2
Defocus range (μm)	0.8-1.8	0.8-1.8
Pixel size (Å)	0.647	0.647
Symmetry imposed	C3	C1
Initial particle images (no.)	1290705	--
Final particle images (no.)	291461	386950
Map resolution (Å)		
FSC threshold (0.143)	3.26	3.6
Map resolution range (Å)		3.5-5.76
**Refinement**		
Initial model used (PDB code)		7LRT
Model resolution (Å)		3.5
FSC threshold		
Model resolution range (Å)		235-3.5
Map sharpening *B* factor (Å^2)		-141
Model composition		
Non-hydrogen atoms		3239
Protein residues		411
Ligands		NAG:1
B factors (Å^2)		
Protein		45.4/99.5/72.8
Ligand		92.8/92.8/92.8
R.m.s. deviations		
Bond lengths (Å)		0.004
Bond angles (°)		0.738
Validation		
MolProbity score		2.17
Clashscore		18.79
Poor rotamers (%)		0.88
Ramachandran plot		
Favored (%)		93.98
Allowed (%)		6.02
Disallowed (%)		0
