# Supplementary material for: Computationally restoring the potency of a clinical antibody against Omicron
Source: Nature. 2024 May 8;629(8013):878–85. doi: 10.1038/s41586-024-07385-1 (PMC11111397; doi:10.1038/s41586-024-07385-1)
Supplement: Supplementary file 2 — Reporting Summary [file 41586_2024_7385_MOESM2_ESM.pdf]

nature portfolio

Corresponding author(s): Thomas A. Desautels, Daniel M. Faissol

Last updated by author(s): Mar 13, 2024

Reporting Summary

Nature Portfolio wishes to improve the reproducibility of the work that we publish. This form provides structure for consistency and transparency in reporting. For further information on Nature Portfolio policies, see our Editorial Policies and the Editorial Policy Checklist.

Statistics

For all statistical analyses, confirm that the following items are present in the figure legend, table legend, main text, or Methods section.

n/a	Confirmed	
☐	☒	The exact sample size (*n*) for each experimental group/condition, given as a discrete number and unit of measurement
☐	☒	A statement on whether measurements were taken from distinct samples or whether the same sample was measured repeatedly
☐	☒	The statistical test(s) used AND whether they are one- or two-sided<br>*Only common tests should be described solely by name; describe more complex techniques in the Methods section.*
☒	☐	A description of all covariates tested
☐	☒	A description of any assumptions or corrections, such as tests of normality and adjustment for multiple comparisons
☐	☒	A full description of the statistical parameters including central tendency (e.g. means) or other basic estimates (e.g. regression coefficient) AND variation (e.g. standard deviation) or associated estimates of uncertainty (e.g. confidence intervals)
☐	☒	For null hypothesis testing, the test statistic (e.g. *F*, *t*, *r*) with confidence intervals, effect sizes, degrees of freedom and *P* value noted<br>*Give P values as exact values whenever suitable.*
☒	☐	For Bayesian analysis, information on the choice of priors and Markov chain Monte Carlo settings
☒	☐	For hierarchical and complex designs, identification of the appropriate level for tests and full reporting of outcomes
☒	☐	Estimates of effect sizes (e.g. Cohen's *d*, Pearson's *r*), indicating how they were calculated

Our web collection on statistics for biologists contains articles on many of the points above.

Software and code

Policy information about availability of computer code

Data collection	Biological data collection was conducted with appropriate, instrument specific software.
Data analysis	Analysis of individual experiments was conducted in GraphPad Prism v.8.1, 9.0, or 10.2, as noted in individual figure legends.<br><br>Protein Thermal Shift ™ software version 1.4 (ThermoFisher) was used to generate the melting temperature and fit data.<br><br>Antibody-antigen structures were minimized and relaxed using standard minimization procedures from Rosetta, Chimera, and GROMACS.<br><br>The ImprovWF dynamic workflows code is available at:<br>https://github.com/LLNL/improvwf<br><br>Selected GUIDE workflow components are available at:<br>https://github.com/LLNL/CRPCA<br><br>Antibody-antigen minimization procedures used Rosetta 3.13, Chimera v1.15, or GROMACS v2021.4<br><br>Rosetta FlexDDG. Script collected from github in 2020:<br>https://github.com/Kortemme-Lab/flex_ddG_tutorial/blob/master/analyze_flex_ddG.py<br><br>SFE v1.0

ddG estimates calculated with FoldX v4 or Rosetta 3.13

All DMS data analysis was performed using dms-vep-pipeline version 1.8, which can be accessed at https://github.com/dms-vep/dms-vep-pipeline/tree/51e73d601bd770eb6e9abd21f57fb4365699c984

Code and notebooks related to DMS runs are available at:
https://dms-vep.github.io/SARS-CoV-2_Omicron_BA.1_spike_DMS_COV2-2130/
https://dms-vep.github.io/SARS-CoV-2_Omicron_BA.2_spike_DMS_COV2-2130/

Pseudocode for additional components is available in the Supplemental Material.

For manuscripts utilizing custom algorithms or software that are central to the research but not yet described in published literature, software must be made available to editors and reviewers. We strongly encourage code deposition in a community repository (e.g. GitHub). See the Nature Portfolio guidelines for submitting code & software for further information.

Data

Policy information about availability of data

All manuscripts must include a data availability statement. This statement should provide the following information, where applicable:

- Accession codes, unique identifiers, or web links for publicly available datasets
- A description of any restrictions on data availability
- For clinical datasets or third party data, please ensure that the statement adheres to our policy

The EM map and model has been deposited into EMDB (EMD-28198, EMD-28199) and PDB (8EKD). Other protein structural data were employed in this work (PDB: 7L7E, 7T9K) and in analysis (PDB: 7X66, 7XAZ , 8IOS, 8IF2, 8GB8). Sequence data that support the findings of this study have been deposited in GenBank under accessions PP474664 – PP474679 and are available in the Supplementary Information. Source data for Figure 4 are provided with the paper. DMS library variant data and antibody per replicate DMS selection data can be accessed at https://github.com/dms-vep/SARS-CoV-2_Omicron_BA.2_spike_DMS_COV2-2130 and https://github.com/dms-vep/SARS-CoV-2_Omicron_BA.1_spike_DMS_COV2-2130 GitHub repositories.

Human research participants

Policy information about studies involving human research participants and Sex and Gender in Research.

Reporting on sex and gender	N/A
Population characteristics	N/A
Recruitment	N/A
Ethics oversight	N/A

Note that full information on the approval of the study protocol must also be provided in the manuscript.

Field-specific reporting

Please select the one below that is the best fit for your research. If you are not sure, read the appropriate sections before making your selection.

☒ Life sciences ☐ Behavioural & social sciences ☐ Ecological, evolutionary & environmental sciences

For a reference copy of the document with all sections, see nature.com/documents/nr-reporting-summary-flat.pdf

Life sciences study design

All studies must disclose on these points even when the disclosure is negative.

Sample size	For animal studies, no sample sizes were chosen a priori but instead estimated based on prior knowledge of anticipated experimental differences among groups. All experiments with statistical analysis were repeated at least two independent times, each with multiple technical replicates. Experimental size of animal cohorts was determined based on prior experience performing studies in mice.
Data exclusions	Representative data were chosen where appropriate in lieu of combining independent replicates. Data were excluded only for technical reasons (e.g., splash into wells). No data was excluded from animal studies.
Replication	All replications executed were consistent with the representative data presented in the manuscript. Each DMS experiment was done using two independently produced deep-mutational scanning libraries. For animals studies, all experiments had multiple biological and/or technical replicates and are indicated the Figure legends.
Randomization	For animal studies, mice were randomly assigned from large batches obtained from the vendor to different experimental groups in an age-

Randomization	matched distribution.
Blinding	For animal studies, no blinding was performed as handling of BSL3 virus requires exact tracking of infected mice and samples.

Behavioural & social sciences study design

All studies must disclose on these points even when the disclosure is negative.

Study description	*Briefly describe the study type including whether data are quantitative, qualitative, or mixed-methods (e.g. qualitative cross-sectional, quantitative experimental, mixed-methods case study).*
Research sample	*State the research sample (e.g. Harvard university undergraduates, villagers in rural India) and provide relevant demographic information (e.g. age, sex) and indicate whether the sample is representative. Provide a rationale for the study sample chosen. For studies involving existing datasets, please describe the dataset and source.*
Sampling strategy	*Describe the sampling procedure (e.g. random, snowball, stratified, convenience). Describe the statistical methods that were used to predetermine sample size OR if no sample-size calculation was performed, describe how sample sizes were chosen and provide a rationale for why these sample sizes are sufficient. For qualitative data, please indicate whether data saturation was considered, and what criteria were used to decide that no further sampling was needed.*
Data collection	*Provide details about the data collection procedure, including the instruments or devices used to record the data (e.g. pen and paper, computer, eye tracker, video or audio equipment) whether anyone was present besides the participant(s) and the researcher, and whether the researcher was blind to experimental condition and/or the study hypothesis during data collection.*
Timing	*Indicate the start and stop dates of data collection. If there is a gap between collection periods, state the dates for each sample cohort.*
Data exclusions	*If no data were excluded from the analyses, state so OR if data were excluded, provide the exact number of exclusions and the rationale behind them, indicating whether exclusion criteria were pre-established.*
Non-participation	*State how many participants dropped out/declined participation and the reason(s) given OR provide response rate OR state that no participants dropped out/declined participation.*
Randomization	*If participants were not allocated into experimental groups, state so OR describe how participants were allocated to groups, and if allocation was not random, describe how covariates were controlled.*

Ecological, evolutionary & environmental sciences study design

All studies must disclose on these points even when the disclosure is negative.

Study description	*Briefly describe the study. For quantitative data include treatment factors and interactions, design structure (e.g. factorial, nested, hierarchical), nature and number of experimental units and replicates.*
Research sample	*Describe the research sample (e.g. a group of tagged Passer domesticus, all Stenocereus thurberi within Organ Pipe Cactus National Monument), and provide a rationale for the sample choice. When relevant, describe the organism taxa, source, sex, age range and any manipulations. State what population the sample is meant to represent when applicable. For studies involving existing datasets, describe the data and its source.*
Sampling strategy	*Note the sampling procedure. Describe the statistical methods that were used to predetermine sample size OR if no sample-size calculation was performed, describe how sample sizes were chosen and provide a rationale for why these sample sizes are sufficient.*
Data collection	*Describe the data collection procedure, including who recorded the data and how.*
Timing and spatial scale	*Indicate the start and stop dates of data collection, noting the frequency and periodicity of sampling and providing a rationale for these choices. If there is a gap between collection periods, state the dates for each sample cohort. Specify the spatial scale from which the data are taken*
Data exclusions	*If no data were excluded from the analyses, state so OR if data were excluded, describe the exclusions and the rationale behind them, indicating whether exclusion criteria were pre-established.*
Reproducibility	*Describe the measures taken to verify the reproducibility of experimental findings. For each experiment, note whether any attempts to repeat the experiment failed OR state that all attempts to repeat the experiment were successful.*
Randomization	*Describe how samples/organisms/participants were allocated into groups. If allocation was not random, describe how covariates were controlled. If this is not relevant to your study, explain why.*
Blinding	*Describe the extent of blinding used during data acquisition and analysis. If blinding was not possible, describe why OR explain why blinding was not relevant to your study.*

Did the study involve field work? ☐ Yes ☐ No

Field work, collection and transport

Field conditions	*Describe the study conditions for field work, providing relevant parameters (e.g. temperature, rainfall).*
Location	*State the location of the sampling or experiment, providing relevant parameters (e.g. latitude and longitude, elevation, water depth).*
Access & import/export	*Describe the efforts you have made to access habitats and to collect and import/export your samples in a responsible manner and in compliance with local, national and international laws, noting any permits that were obtained (give the name of the issuing authority, the date of issue, and any identifying information).*
Disturbance	*Describe any disturbance caused by the study and how it was minimized.*

Reporting for specific materials, systems and methods

We require information from authors about some types of materials, experimental systems and methods used in many studies. Here, indicate whether each material, system or method listed is relevant to your study. If you are not sure if a list item applies to your research, read the appropriate section before selecting a response.

Materials & experimental systems

n/a	Involved in the study	
☐	☒	Antibodies
☐	☒	Eukaryotic cell lines
☒	☐	Palaeontology and archaeology
☐	☒	Animals and other organisms
☒	☐	Clinical data
☒	☐	Dual use research of concern

Methods

n/a	Involved in the study	
☒	☐	ChIP-seq
☒	☐	Flow cytometry
☒	☐	MRI-based neuroimaging

Antibodies

Antibodies used

Gyrolab immunoassays: A secondary detection antibody served as a fluorescent reporter: Alexa Fluor 647 AffiniPure Fab Fragment Goat Anti-Human IgG, Fcγ fragment specific (Jackson ImmunoResearch). The detection Fab was used at 100 nM
Our bridging control antibody was s309 (Biovision, A2266 -100) - https://www.abcam.com/products/primary-antibodies/sars-cov-2-spike-glycoprotein-s1-antibody-s309-bsa-and-azide-free-ab289796.pdf
Positive control for pseudovirus neutralization assay: S2K146 (DOI: 10.1126/science.abm8143)
Negative control for pseudovirus and authentic virus neutralization assays: DENV-2D22 (doi:10.1073/pnas.1200566109)
ELISA: 1:5000 dilution goat anti-human IgG conjugated with horseradish peroxidase (HRP) (Southern Biotech, cat. 2014-05, lot L2118-VG00B)
FRNT: Anti-S murine antibodies (PMID: 34481543) Dilution: N/A This pool of antibodies was used as pooled hybridoma supernatant
FRNT: HRP-conjugated goat anti-mouse IgG (Sigma Cat # A8924, RRID: AB_258426) 1:1000 dilution
Mouse studies isotype control antibody: anti-West Nile hE16 mAb (Oliphant2005, PMID: 15852016)
Reagent antibodies were obtained from Jackson Immunoresearch 109-607-008

Validation

Validation of all primary antibodies tested for binding antigen by ELISA or with infected cells.
For validation data on commercial antibodies used, refer to specification documents from the manufacturers as follows:
HRP-conjugated Goat anti-mouse IgG - https://www.sigmaaldrich.com/specification-sheets/638/581/A8924-BULK.pdf
HRP-conjugated Goat anti-human IgG, Southern Biotech, cat. 2014-05, lot L2118-VG00B, https://resources.southernbiotech.com/techbul/2014.pdf
AlexaFluor647-conjugated Goat Anti-Human IgG, $F(ab')_2$ - https://www.jacksonimmuno.com/lots/000000168261

Eukaryotic cell lines

Policy information about cell lines and Sex and Gender in Research

Cell line source(s)

HEK293 cells at ATUM (Newark, CA, USA)
ExpiCHO-S™ Cells (Thermofisher scientific Cat. No. A29127)
Expi293F™ Cells (Thermofisher scientific Cat. No. A14527
HEK293T (ATCC: CRL-11268)
Vero-hACE2-TMPRSS2 cells in FRNT were generated at the NIH (VRC, Barney Graham lab) now BEI (BEI NR-54970) - (PMID: 32404436)

	VAT cells used in Plaque assays: BEI Resources, NIAID, NIH<br>DMS experiments were performed using 293T-hACE2 cells available from BEI:NR-52511
Authentication	None authenticated.
Mycoplasma contamination	All cell lines were confirmed to be negative for mycoplasma during a regular basis.
Commonly misidentified lines<br>(See ICLAC register)	None.

Animals and other research organisms

Policy information about studies involving animals; ARRIVE guidelines recommended for reporting animal research, and Sex and Gender in Research

Laboratory animals	Seven to nine-week-old female heterozygous 18-hACE2 C57BL/6J mice (strain: 2B6.Cg-Tg(K18-ACE2)2Prlmn/J, Cat # 34860) were obtained from The Jackson Laboratory. Mice were housed in groups of 3 to 5. Photoperiod = 12 hr on:12 hr off dark/light cycle. Ambient animal room temperature is 70° F, controlled within ±2° and room humidity is 50%, controlled within ±5%.
Wild animals	No wild animals were used in this study.
Reporting on sex	Only female mice were used in the study.
Field-collected samples	No field collected samples were used in this study.
Ethics oversight	The protocols were approved by the Institutional Animal Care and Use Committee at the Washington University School of Medicine (assurance number A3381–01).

Note that full information on the approval of the study protocol must also be provided in the manuscript.
